# Green synthesis, biological and molecular docking of some novel sulfonamide thiadiazole derivatives as potential insecticidal against *Spodoptera littoralis*

**DOI:** 10.1038/s41598-023-46602-1

**Published:** 2023-11-06

**Authors:** Ahmed M. El-Saghier, Souhaila S. Enaili, Asmaa M. Kadry, Aly Abdou, Mohamed A. Gad

**Affiliations:** 1 Chemistry Department, Faculty of Science, Sohag University, Sohag, 282524 Egypt; 2 Chemistry Department, Faculty of Science, Al Zawiya University, Al Zawiya, Libya; 3 Research Institute of Plant Protection, Agricultural Research Center, Giza, 12619 Egypt

**Keywords:** Biochemistry, Biological techniques, Environmental sciences, Chemistry, Materials science

## Abstract

Although crop plants provide the majority of human food, pests and insects frequently cause huge economic losses. In order to develop innovative insecticidal compounds with low toxicity and a positive environmental impact, we developed new *N*-(4-sulfamoylphenyl)-1,3,4-thiadiazole-2-carboxamide derivatives (**2–12**). With the use of spectroscopic techniques and elemental data, the chemical structure of these new compounds was meticulously clarified. The toxicological and biological effects of the synthesized compound of the cotton leafworm *Spodoptera littoralis* (Boisduval, 1833) under laboratory conditions were also investigated. Regarding the determined LC_50_ values, compounds **3, 7, 8**, and **10** showed the most potent toxic effect with LC_50_ values of 29.60, 30.06, 27.65 and 29.01 ppm, respectively. A molecular docking investigation of twelve synthetic compounds (from compound **2** to compound **12**) was performed against AChE (Acetylcholinesterase). There was a wide range of binding affinities shown by these compounds. This work suggests that these substances may have insecticidal and AChE inhibitory properties, and it may be possible to further explore them in the process of creating pesticides that target AChE.

## Introduction

**Figure 1:**
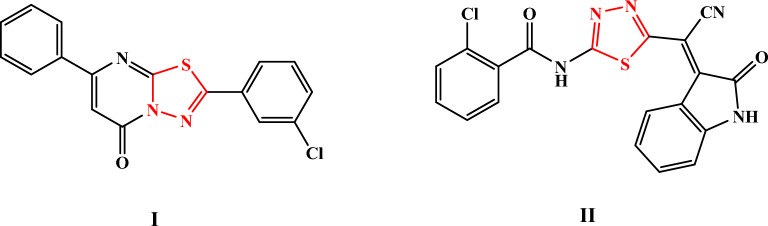
1,3,4-thiadiazole derivatives as insecticide against *Spodoptera littoralis*.

Sulfonamides, a chemical family of sulfur-containing insecticides, have recently attracted considerable interest for their ability to modulate the features of novel crop protection chemicals. It is well known that 1,3,4-thiadiazoles and their derivatives exhibit a variety of biological actions, not just in studies on medicines as antimicrobial^1^, anticancer^2^ agents, ant tuberculosis^3^, anti-inflammatory activities^4^, antiviral^5^, or anticonvulsant^6^, but also in research on pesticides as antifungal^7^, insecticidal and also as plant growth regulators^8^. For the development of new medications, heterocyclic chemistry is essential since many heterocyclic molecules, such as “1,3,4-thiadiazole,” are therapeutically active. Omar et al. produced 1,3,4-thiadiazole-ringed *N,N*-disubstituted piperazine compounds and evaluated their antibacterial and antifungal activity. In order to corroborate the activity, they also carried out molecular docking experiments. Because compound 1,3,4-thiadiazole analogues effectively reduced bacterial and fungal stains more effectively than the industry standard, they came to the conclusion that it was highly potent. During docking experiments, the substance also demonstrated correct interaction with a decent binding score, making it a strong candidate for anti-microbial efficacy^9^. Although crops are the main source of food for humans, crop losses caused by insects result in considerable annual economic losses. Chemical pesticides are still the main method for controlling them, but their use raises concerns for the environment and poses risks to both human and animal health. In addition, they can lead to the development of insecticide resistance. As a result, long-lasting and affordable alternative pest control methods have been consistently developed. In order to find new pesticides, research into the synthesis and bioassays of 1,3,4-thiadiazole derivatives has been increasingly popular. Utilizing several electrophilic reagents, researchers created the new 1,3,4-thiadiazole analogues **I** and **II** various spectroscopic techniques were used to establish their structural integrity, Fig. 1^10^. Using the leaf dip method, the synthesized substances were then evaluated for in-vitro insecticidal efficacy against (cotton leaf worm) *S. littoralis* larvae.

Acetylcholinesterase (AChE) inhibitors are a class of drugs that can enhance the levels of acetylcholine, a neurotransmitter involved in memory and cognition, by preventing its breakdown by the enzyme AChE. Various class of compounds have been newly reported as an acetyl cholinesterase inhibitors^11–13^. AChE inhibitors are used to treat Alzheimer’s disease, a neurodegenerative disorder characterized by progressive cognitive decline and memory loss. Synthesized AChE inhibitors are compounds that are designed and produced in the laboratory, based on the structure and activity of natural or existing AChE inhibitors. Some examples of synthesized AChE inhibitors are: Pyridoxine–triazoles: These are hybrid molecules that combine pyridoxine, a natural product and a precursor of vitamin B6, with triazole, a heterocyclic ring that can bind to the active site of AChE. These compounds showed potent AChE inhibition, antioxidant and metal chelation properties in vitro^14^. XJP-1: This is a novel compound that was derived from tacrine, a first-generation AChE inhibitor. XJP-1 showed improved AChE inhibition and reduced amyloid plaque formation in vivo, using a transgenic Drosophila model of Alzheimer’s disease^15^. Quinoxaline derivatives: These are compounds that contain a quinoxaline ring, which is similar to the benzimidazole ring of donepezil, a second-generation AChE inhibitor. These compounds exhibited selective and reversible AChE inhibition and good blood–brain barrier permeability in silico^16^. Isoindolone derivatives: These are compounds that contain an isoindolone ring, which is similar to the indanone ring of rivastigmine, another second-generation AChE inhibitor. These compounds demonstrated enhanced AChE inhibition and antioxidant activity in vitro^17^. Synthesized AChE inhibitors are promising candidates for the development of new drugs for Alzheimer’s disease, as they can target multiple aspects of the disease pathogenesis and offer better efficacy and safety profiles than the currently available drugs. A species of moth of the Noctuidae family called *Spodoptera littoralis* (Boisduval, 1833) may be found all throughout Africa, Mediterranean Europe, and the Middle The cotton leaf worm is well recognized to cause significant financial losses for many nations^18,19^. The exceedingly hazardous *S. littoralis* polyphosphorous moth consumes more than 100 types of valuable commercial plants, such as cotton, potatoes, maize and vegetables^20^. For the aforementioned reasons as well as to continue our program in the synthesis of physiologically active heterocyclic compounds to repel this insect, the authors were interested in developing novel, environmentally safe East. Insecticidal chemicals with little toxicity^21–23^. In this various work we used 2-hydrazinyl-*N*-(4-sulfamoylphenyl)-2-thioxoacetamide^24^, allowed to react with various aldehydes to create novel *N*-(4-sulfamoylphenyl)-1,3,4-thiadiazole-2-carboxamide derivatives. Additionally, the cotton leafworm *S. littoralis* larvae of the 2nd and 4th larvae instar were used to investigate the insecticidal activity of the synthetic compounds.

## Results and discussions

### Synthesis

**Figure 2:**
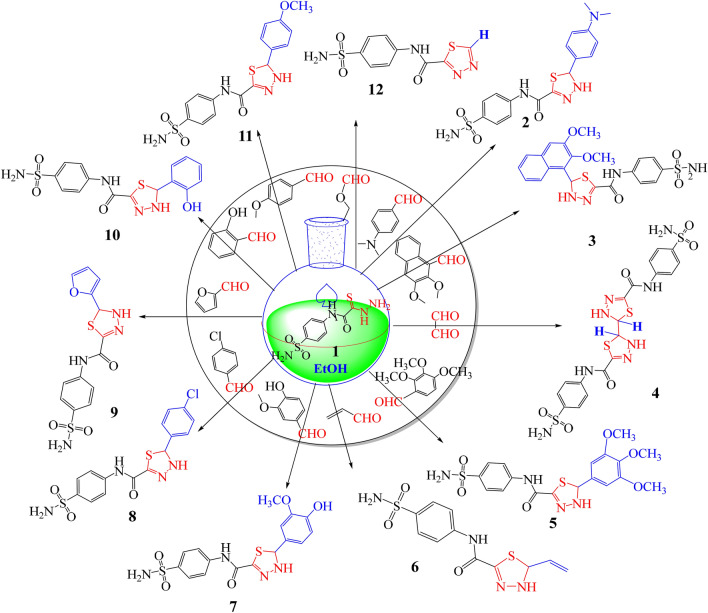
Designing of novel Sulfonamide-thiadiazole derivatives (**2–12**).

Our approach is to figure out how to use these compounds as building blocks for the synthesis of various five, six, and seven-membered rings as a continuation of our work on the synthesis of heterocycles^25–32^. Herein, we interest to produce a new and not reported green method to synthesis a series of new *N*-(4-sulfamoylphenyl)-1,3,4-thiadiazole-2-carboxamide derivatives **2–12**, Fig. 2.

**Figure 3:**
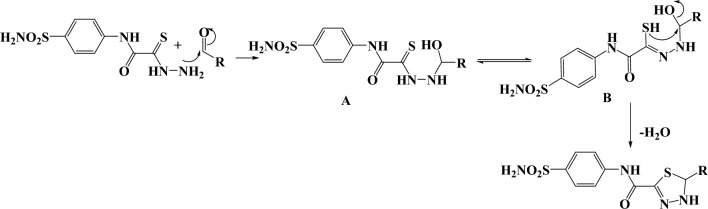
Chemical synthesis of 2,5-disubstituted-1,3,4-thiadiazole derivatives.

2-Hydrazinyl-*N*-(4-sulfamoylphenyl)-2-thioxoacetamide **(1)**^24^ reacted with a series of different aldehydes in ethanol under reflux for about 2 h, without any catalyst, see Fig. 2. The reaction mechanism for preparation of a novel 2,5-disubstituted-1,3,4-thiadiazole derivatives **2–12** was assumed to proceed via a nucleophilic attack of NH_2_ group of thiohydrazide at the carbonyl carbon of aldehyde to afford the intermediate **A** which tautomerize to the thiol form **B** and attack at the C–OH followed by elimination of water molecule ( as well as ethanol molecule in case of compounds **12**) to afford the thiadiazole moiety, Fig. 3.

The structures of these synthesized compounds were determined using FT-IR, ^1^H NMR, ^13^C NMR spectroscopy, and elemental analysis spectroscopic methods. The IR spectrum of compounds **2–12** revealed the disappearance of NH, NH_2_ groups of thiohydrazide and appearance of new bands at 1650–1684 cm^−1^ belonged to carbonyl group, new bands at 3350–3190 cm^−1^ related to the OH groups for compounds** 7** and **10**, and appearance of a new bands at 3050–3079 cm^−1^ which belongs to aromatic groups and also appearance of a new bands at 2880–2980 cm^−1^ assigned to aliphatic groups in compounds. The ^1^HNMR spectra showed signals between 10.50 and 9.80 ppm belong to amidic NH groups (disappearance by D_2_O), between 9.60 and 9.10 ppm refer to the NH groups of thiadiazole rings (disappearance by D_2_O) and CH signals appear between 7.20 and 5.60 ppm due to the formation of thiadiazole rings. The ^13^CNMR confirmed the expected structure by appearance of new signals between 56.50 and 76.60 ppm owing to CH of thiadiazole nucleus. Moreover, elemental analysis spectroscopic methods obtained information about the elemental composition of synthesized compounds.

### Toxicological studies

#### Toxicological effectiveness checking for 2nd larvae

**Figure 4:**
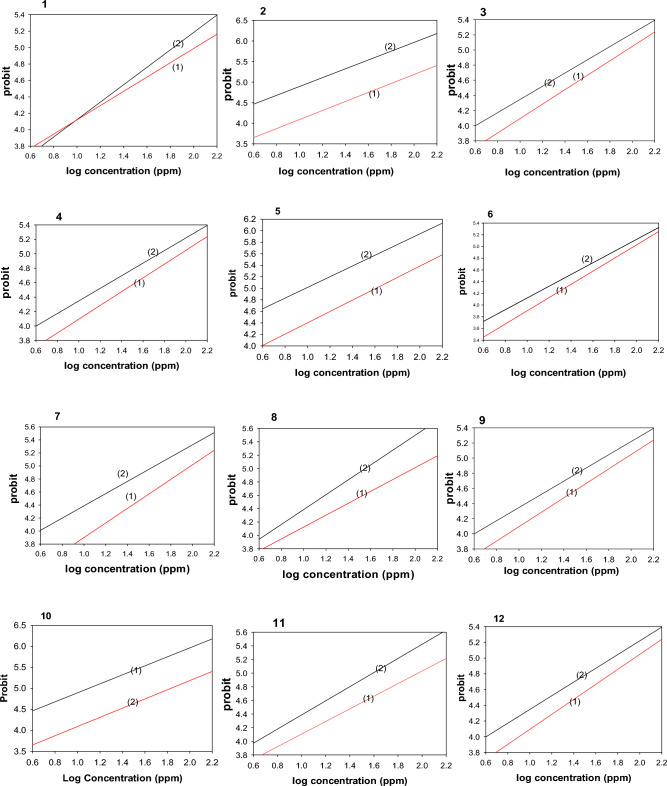
Insecticidal effectiveness of selective compounds **1–12** against 2nd and 4th instar larvae of *S. littoralis*.

Table 1 and Fig. 4 shows the results of tests done on target compounds **1** through **12** on *S. littoralis* insect 2nd instar larvae. The LC_50_ values of the investigated insecticidal bioefficacy against the second larvae instar range from strong to low toxicological activity, with LC_50_ values varied between 29.60 and 96.66 ppm. Aside from that, the LC_50_ values for compounds **1** through **12** were 88.68, 43.49, 29.60, 92.58, 33.17 , 95.37, 30.06, 27.65, 38.00, 29.01, 31.02, and 96.66 ppm, respectively, while the toxicity index values were 31.17, 63.57, 83.35, 29.86, 93.41, 28.99, 90.18, 100, 72.76, 95.31, 89.13 and 28.60%. In light of the calculated LC_50_ values of sulfonate bearing the thiadiazole moiety, **8, 10, 3, 7** and **11** demonstrated the most potent toxic effect with LC_50_ values of 27.65, 29.01, 29.60, 30.06 and 31.02 ppm, respectively.

**Table 1:** Insecticidal effectiveness of components **1–12** toward the 2nd and 4th larvae instar of *S. littoralis* after 3 days of treatment.

2nd instar larvae					4th instar larvae			
Comp	LC_50_ (ppm)	Slope	Toxic ratio^a^	χ^2^	LC_50_ (ppm)	slope	Toxic ratio	χ^2^
**1**	88.68	0.480 ± 0.244	31.17	0.584	133.53	0.613 ± 0.248	39.94	0.285
**2**	43.49	0.564 ± 0.244	63.57	0.425	131.01	0.581 ± 0.247	40.71	0.140
**3**	29.60	0.688 ± 0.248	93.41	0.395	57.58	0.564 ± 0.244	92.63	0.174
**4**	92.58	0.724 ± 0.249	29.86	0.596	152.36	0.626 ± 0.240	35.00	0.159
**5**	33.17	0.691 ± 0.267	83.35	0.924	120.40	0.640 ± 0.250	44.30	0.036
**6**	95.37	0.664 ± 0.248	28.99	0.569	155.69	0.668 ± 0.252	34.26	0.198
**7**	30.06	0.739 ± 0.268	90.18	0.660	106.58	0.636 ± 0.248	49.92	0.124
**8**	27.65	0.751 ± 0.250	100	0.650	53.34	0.622 ± 0.245	100	0.063
**9**	38.00	0.748 ± 0.268	72.76	0.748	140.02	0.583 ± 0.248	38.09	0.004
**10**	29.01	0.781 ± 0.270	95.31	0.312	89.61	0.692 ± 0.248	59.52	0.610
**11**	31.028	0.697 ± 0.267	89.13	1.180	109.49	0.668 ± 0.249	48.71	0.027
**12**	96.66	0.540 ± 0.245	28.60	0.343	151.45	0.618 ± 0.249	35.21	0.116

Toxicity Ratio is calculated as less LC_50_ value for baseline toxicity/the compounds’ LC_50_ value.

#### Toxicological effectiveness checking for adults 4th larvae

Table 1 shows the results of tests done on target compounds **1** through **12** on *S. littoralis* insect 2nd instar larvae. The LC_50_ values of the investigated insecticidal bio efficacy against the fourth larvae instar range from strong to low toxicological activity, with LC_50_ values varied between 53.34 and 151.45 ppm. Aside from that, the LC_50_ values for compounds **1** through **12** were 133.53, 131.01, 57.58, 152.36, 120.40, 155.69, 106.58, 53.34, 140.02, 89.61, 109.61 and 151.45 ppm, respectively, while the toxicity index values were 39.94, 40.71, 92.63, 35.00, 44.30, 34.26, 49.92, 100, 38.09, 59.52, 48.71 and 35.21%. In light of the calculated LC_50_ values of sulfonamide bearing the thiadiazole moiety, **8, 3, 10, 7** and **11** demonstrated the most potent toxic effect with LC_50_ values of 53.34, 57.58, 89.61, 106.58 and 109.49 ppm, respectively.

### Biological studies

Numerous biological characteristics of *S. littoralis* are being examined for their effects of the synthetic Target components **8, 10, 3,** and **7**. Recently moulted *S. littoralis* fourth instar larvae were fed caster bean leaves treated with LC_25_ concentrations of the most lethal sulfonamide thiadiazole derivatives **8, 10, 3**, and **7** for 48 h before being switched to untreated leaves until pupation as part of an investigation into the biological traits of the species. After presenting the crucial biological aspects, Tables 2 and 3 exhibit the findings.

**Table 2:** The very freshly prepared target components **3, 7, 8** and **10** had biological effect against of *S. littoralis* larvae instar at their LC_25_ values under laboratory conditions.

Tested compound	LC25 ppm	Larval duration Days ± SE	Pupal duration Days ± SE	weight (mg) ± SE	Normal pupae % ± SE	Deformed pupae % ± SE	Adult emergence % ± SE
**3**	8.29	17.06^c^ ± 0.01	11.20^d^ ± 0.01	282.60^c^ ± 0.14	83.25^c^ ± 0.35	9.55^c^ ± 0.30	60.31^c^ ± 0.55
**7**	10.02	14.35^d^ ± 0.20	17.03^b^ ± 0.20	280.15^b^ ± 0.14	87.25^b^ ± 0.80	9.40b^c^ ± 0.20	81.24^b^ ± 0.32
**8**	6.70	22.61^a^ ± 0.20	10.22^c^ ± 0.20	265.71^e^ ± 0.20	38.56^e^ ± 0.56	17.23^a^ ± 0.34	71.35^d^ ± 0.50
**10**	7.88	19.33^b^ ± 0.01	11.65^d^ ± 0.20	274.12^d^ ± 0.18	71.51^d^ ± 0.40	15.32^b^ ± 0.33	60.23^c^ ± 0.52
Control	-	10.51^e^ ± 0.20	19.30^a^ ± 0.20	292.10^a^ ± 0.28	93.21^a^ ± 0.29	3.20^d^ ± 0.17	92.41^a^ ± 0.62

Letters mean the noteworthy differences between treatments in line with Duncan’s check SE = Standard error.

**Table 3:** The effect of **3, 7, 8** and **10** components on the fecundity, fertility and adult longevity for *S. littoralis* under laboratory conditions.

Tested compound	No. of eggs/female ± SE	Fecundity% ± SE	Egg hatchability% ± SE
**3**	1320.24^c^ ± 19.36	66.35^c^ ± 0.02	65.17^b^ ± 0.30
**7**	1908.64^b^ ± 11.20	78.23^b^ ± 0.02	74.20^b^ ± 0.25
**8**	802.39^d^ ± 14.58	23.85^e^ ± 0.20	44.32^d^ ± 0.21
**10**	906.32^d^ ± 10.25	44.60^d^ ± 0.1	52.23^c^ ± 0.04
Control	2905.52^a^ ± 13.5	100^a^	98.24^a^ ± 0.29

Letters mean the noteworthy differences between treatments in line with Duncan’s check SE = Standard error.

#### Larval and pupal duration

All of the tested substances significantly increased the larval duration, which was **8** (22.16 days), **10** (19.33 days), **3** (17.06 days), and **7** (14.53 days), respectively, compared to the control group (10.5 days), as shown in Table 2. The LC_25_ values of compounds **8, 10, 3,** and** 7** were 6.70, 7.88, 8.29, and 10.02 ppm. In contrast, the tested components decreased the pupal period with statistically significant differences from one another, tabulating as **8** (10.22 days) and **10** (11.65 days), while **3** and** 7** had no significant differences, tabulating as (11.20 and 17.03) days, respectively, in comparison to the untreated larvae (19.30 days).

#### Pupal weight

The results listed in Table 2 demonstrate that the pupal weight trended in the same direction. In comparison to the control pupal weight of 92.10 mg, all of the components under study considerably reduced pupal weight. Component **8** was the most effective, recording 265.71 mg, followed by components **3**, **10**, and **7** at 274.12, 282.60 2, and 280.15 395.14 mg, respectively.

#### % of normal, deformed pupae and adult emergency

As can be seen in Table 2, the latent effects **8, 10, 3,** and** 7** recorded the highest value of malformed pupae, healthy pupae, and adult emergence, recording (71.51, 38.56, 83.25, and 87.25%), (17.23, 15.32, 9.55, and 9.405%), and (71.35, 60.23, 60.31, and 81.24%), respectively, compared to the control (93.21, 3.20, 92%).

#### % of Fecundity and Egg hatchability

Compounds **8** and **10** have dramatically decreased fecundity, as shown by Table 3 findings regarding the average number of eggs laid by adult females (fecundity), the fecundity rate, and the hatchability rate. In the other hand, after treatment of the parent fourth instar larvae, eggs hatchability (fertility) was abruptly reduced in the offspring generation, with **8** recording 802.39 eggs per female, 23.85 fecundity, and 44.32% eggs hatchability, followed by 10 (906.32 eggs per female, 44.60 fecundity, and 52.23% eggs hatchability), in contrast to the control group (2905 eggs per female, 100 fecundity, and eggs hatchability was 98.24%. The least fertile compound, compound **3**, had 1320 eggs per female, a fecundity of 66.35%, and a fertility of 65.17%, whereas compound **7** had 1908.64% eggs per female, a fecundity of 78.23%, and a fertility of 74.20%.

### Molecular docking

Molecular docking is a method that predicts the preferred orientation and binding affinity of one molecule (ligand) to another molecule (receptor) when they form a stable complex^33,34^. Molecular docking is important for understanding the molecular interactions that underlie biological processes such as signal transduction, enzyme catalysis, and drug action^35,36^. Molecular docking is also widely used in structure-based drug design, as it can help identify potential drug candidates that bind to a specific target protein^37,38^. First, the re-docking and superimposition methods were used to validate the docking operation^35^. The 2ACE’s natural ligand was taken out and docked back into the active site. Re-docking was done to evaluate the efficiency of the docking process. The re-docking procedure followed the same methods as it did for the studied chemicals. In the re-docking validation stage, the binding pattern of the co-crystallized ligand was successfully recreated, demonstrating that the docking procedure used was suitable for the desired docking inquiry. Figure S37 displays the superimposition of the re-docked ligand and the native co-crystallized one with a small RMSD of 1.012.

The docking scores of the investigated compounds (from compound **2** to compound **12**) against the AChE enzyme (PDB ID 2ACE) are shown in Table 4. The docking scores for the compounds ranged from − 11.08 to − 7.06 kcal/mol. The compound with the greatest score was compound **10** (− 11.08 kcal/mol), followed by compound **8** (− 10.66 kcal/mol), compound **7** (− 10.19 kcal/mol), compound **3** (− 9.54 kcal/mol), compound **11** (− 9.51 kcal/mol), compound **2** (− 8.79 kcal/mol), compound **5** (− 8.60 kcal/mol), compound **9** (− 8.09 kcal/mol), compound **4** (− 7.63 kcal/mol), and compound **6** (− 7.27 kcal/mol), whereas the compound with the lowest score was compound **12** (− 7.06 kcal/mol).

**Table 4:** Docking scores of the investigated compounds (from compound **2** to compound **12**) against AChE (PDB ID: 2ACE).

	Ligand	Receptor	Interaction	Distance	E (kcal/mol)	S (kcal/mol)
**2**	6-ring	TRP 84	pi–pi	3.61	− 1.04	− 8.79
	N 12	PHE 330	H–pi	3.84	− 1.70	
	N 12	HIS 440	H–acceptor	3.32	− 0.40	
	O 9	GLY 441	H–acceptor	3.43	− 0.70	
**3**	O 9	HIS 440	H–acceptor	2.98	− 1.80	− 9.54
	O 8	GLY 441	H–acceptor	3.47	− 1.70	
**4**	O 8	GLU 199	H–acceptor	2.79	− 1.10	− 7.63
	O 13	TYR 121	H–acceptor	2.73	− 0.80	
	6-ring	SER 286	pi–H	4.19	− 1.40	
**5**	6-ring	TRP 279	pi–pi	3.64	− 1.01	− 8.60
	O 9	HIS 440	H–acceptor	2.86	− 1.60	
**6**	6-ring	TRP 279	pi–pi	3.78	− 0.89	− 7.27
	O 8	HIS 440	H–acceptor	3.14	− 3.00	
**7**	O 13	TYR 70	H–acceptor	3.16	− 0.70	− 10.19
	O 25	SER 286	H–acceptor	3.15	− 0.80	
**8**	S 15	ASN 280	H–donor	3.10	− 1.10	− 10.66
	S 15	ASN 280	H–acceptor	3.36	− 0.90	
	6-ring	ASN 280	pi–H	3.64	− 1.20	
	O 9	PHE 288	H–acceptor	2.84	− 3.50	
**9**	O 8	GLU 199	H–acceptor	2.77	− 1.10	− 8.09
	O 13	TYR 121	H–acceptor	2.79	− 0.90	
**10**	O 9	ASP 72	H–acceptor	3.41	− 0.70	− 11.08
	O 8	TYR 121	H–acceptor	2.72	− 2.40	
	N 18	PHE 288	H–acceptor	3.00	− 1.16	
**11**	O 8	TYR 70	H–acceptor	3.23	− 0.60	− 9.51
	O 13	TYR 121	H–acceptor	2.89	− 1.40	
	N 18	HIS 440	H–acceptor	3.40	2.90	
**12**	O 8	ASP 285	H–acceptor	3.28	− 0.70	− 7.06
	O 8	SER 286	H–acceptor	3.02	− 2.50	
	N 12	SER 286	H–acceptor	3.12	− 0.90	
	O 13	PHE 288	H–acceptor	2.98	− 3.80	
	N 16	PHE 331	H–acceptor	3.46	− 0.90	

Figures S38, S39 depict the binding location of the investigated compounds in the active site of 2ACE interaction 3D and 2D, respectively, while, Table 4 lists the docking data. The analysis of the molecular contacts, the compound **10**, three H-acceptor bonds are formed at distances of 3.41, 2.72, and 3.00 Å between O9, O8, and N18 with ASP72, TYR121, and PHE288, respectively, Table 4. In the case of compound **8**, one H-donor bond is formed at distances of 3.10 Å between S15 with ASN280. Moreover, two H-acceptor bonds are formed at distances of 3.36, and 2.84 Å between S15, and O9 with ASN280, and PHE288, respectively. Additionally, one pi-H bond is formed between the 6-ring and ASN280 at a distance of 3.64 Å, Table 4. In the case of compound **7**, two H-acceptor bonds are formed at distances of 3.16, and 3.15 Å between O13, and O25with TYR70, and SER286, respectively, Table 4. In the case of compound **3**, two H-acceptor bonds are formed at distances of 2.98, and 3.47 Å between O9, and O8 with HIS440, and GLY441, respectively, Table 4.

## Material and methods

### Laboratory bioassay screening

Using industry-standard leaf dip bioassay methods^39–43^, all synthesized sulfonamide thiadiazole derivatives were well purified and evaluated for their insecticidal bioactivity. 0.1 g of compounds **1–12** were dissolved in 10 mL of dimethylformamide and then blended with 5 mL of distilled water for the manufacture of the compound stocks to make 1000 ppm. Prior to use, the stocks were stored in a refrigerator. The LC_50_ values for the target compounds were determined after the test results were published. Five different dosages of sulfonamide thiadiazole compounds and 0.1% Tween 80 were used as surfactants. The second and fourth larvae, which were maintained in glass jars weighing five pounds and were around the same size, were fed nine-centimeter-diameter castor bean leaf discs. The discs were then immersed for 10 s in the concentration being tested. With ten larvae each time, each treatment was repeated three times. The castor bean, *Ricinus communis*, also known as the castor oil plant, is a perennial flowering plant species that belongs to the Euphorbiaceae genus of spurge plants^44,45^. It is the solitary species of both the Ricininae subtribe and the monotypic genus Ricinus.. The type of plant from which we obtained the sample and had Prof. Dr. Ayman Hamouda in the Horticulture Research Institute, Agricultural Research Centre, Egypt authenticate its authenticity is the castor bean, *Ricinus communis*, which is already recognized and saved. We further guarantee that all researchers at the Egyptian agricultural research institute have access to this data. We attest to the fact that a voucher sample of this item has been deposited in a public herbarium at Agricultural Research Center in Egypt, which have deposition number is 137/8. We verified that the castor bean leaf used in our study was in accordance with all applicable institutional, national, and international standards and regulations. The area around the Shandaweel research station in Egypt's Sohag governorate is where the castor bean leaf was found.

### Biological studies

Castor bean leaves were used to feed 4th instar larvae after being soaked in LC_25_ of each chemical examined. A determination was made on adult longevity, fecundity, and fertility. The reported approach was used to calculate the fecundity %^46^.

### Statistical analysis

The mortality was normalized using Abbott’s methodology^47^. Utilizing probity analysis, a quantitative examination of the mortality setback line computations was conducted^48^. To strongly mind the Harmfulness Index, sun formulas were applied^49^. A statistical (LDP-line) equation that estimates LC_50_ values with 95% reasonable limits of upper and lower slope was used to estimate the mortality of larval insects.

### Molecular docking

Molecular docking analyses of the compounds were carried out with the help of the MOE (Molecular Operating Environment)^50^. The structures of the compounds (from compound **2** to compound **12**) and the standard ligand (9-(3-Iodobenzylamino)-1,2,3,4-tetrahydroacridine) were optimized to have the lowest energy levels feasible using the MMFF94x force field. The atomic coordinates of the crystal structures of the target enzyme, acetylcholine esterase (AChE) with the PDB ID of 2ACE, were downloaded from the protein databank. Before docking or doing any analysis, the target structures had polar hydrogen atoms added to them, and any accessible water molecules, native ligands, and undesirable chains were eliminated^51^. With regard to the other parameters, the default values were implemented^52,53^. Re-docking and the superimposition approach were used to validate the docking operation. Removed from the 2ACE and re-docked into the active site was the typical ligand (9-(3-Iodobenzylamino)-1,2,3,4-tetrahydroacridine)^54,55^.

## Experimental section

### Chemistry

In Sohag University, Sohag, Egypt melting points were calculated using a Galan-Kamp apparatus. Using a PerkinElmer 2400 LS Series CHN/O analyzer (Cairo University, Giza, Egypt), elemental analyses were carried out on C, H, and N. A PyeUnicam SP3-100 Spectrophotometer was used to collect IR spectra at Sohag University in Sohag, Egypt, using the KBr disc technique (v max, in cm^-1^). The synthesized compounds’ ^1^HNMR (ppm) and ^13^CNMR spectra were captured using the Bruker ADVANCE 400 MHz spectrometer, DMSO and CDCl_3_ were used as the solvents at Sohag University, Sohag, Egypt. Coupling constants were expressed in Hz, while chemical shifts were expressed in ppm. Two runs were used to test the new compounds’ insecticidal efficacy against *S. littoralis* larvae in their second and fourth instar larvae (Table 1 and Fig. 3). The first run used compounds **1–6**, while the second used compounds **7–12**.

#### General procedure of synthesis of *N*-(4-sulfamoylphenyl)-1,3,4-thiadiazole-2-carboxamide derivatives 2–12

A mixture of 2-hydrazinyl-*N*-(4-sulfamoylphenyl)-2-thioxoacetamide **(1)**^24^ (0.001 mol) and (0.001 mol) of aldehyde derivatives was refluxed in ethanol (15 ml) for about 3 h. The reaction was cooled and the solid precipitate was collected by filtration and crystallized from ethanol.

*5-(4-(dimethylamino)phenyl)-N-(4-sulfamoylphenyl)-4,5-dihydro-1,3,4-thiadiazole-2-carboxamide ****(2)***: Orang solid, Yield (82%). Mp. 250–252 °C. FT IR (KBr) ν max cm^−1^: 3333, 3234, 3169 (2NH, NH_2_), 3106 (CH-_arom_.), 2903(CH-_aliph_.), 1677 (C=O_amidic_, st), and 1158 (S=O, st). ^1^H-NMR (DMSO-*d*_*6*_), *δ ppm*: 10.75 (s, 1H, NH_amide_), 9.23 (s, 1H, NH_thiadiazole_), 7.96–7.64 (m, 8H, CH_arom_.); 6.79 (br, 2H, NH_2_); 6.60 (s, 1H, CH_thiadiazole_), 3.03–2.95 (d, 6H, 2CH_3_), ^13^CNMR (DMSO-*d*_*6*_), *δ ppm*: 153.22 (C=O_amidic_), 141.21 (C_thiadiazole_), 140.15, 132.34 (2C_arom_.), 129.92, 127.03 (2C_arom_.), 122.72, 121.14, 116.10, 122.37 (4CH_arom_.), 57.98 (CH_thiadiazole_), and 46.19 (2CH_3_),; C. F.: C_15_H_17_N_5_O_3_S_2_, M.W : 379.45. Elemental Analysis: C, 47.48; H, 4.52; N, 18.46; Found; C, 47.45; H, 4.48; N, 18.55.

*5-(2,3-dimethoxynaphthalen-1-yl)-N-(4-sulfamoylphenyl)-4,5-dihydro-1,3,4-thiadiazole-2-carboxamide**** (3)****:* Canary yellow solid, Yield (90%). Mp. 230–232 °C FT IR (KBr) ν max cm^-1^: 3342, 3256, 3185 (2NH, NH_2_), 3106 (CH-_arom_.), 2998–2962 (CH-_aliph_.), 1684 (C=O_amidic_, st), and 1161 (S=O, st). ^1^H-NMR (DMSO-*d*_*6*_*)*, *δ ppm*: 10.58 (s, 1H, NH_amide_), 9.50 (s, 1H, NH_thiadiazole_), 7.98–7.80 (m, 5H, CH_arom_.), 7.66–7.44 (m, 4H, CH_arom_.); 7.31–7.24 (d, 2H, NH_2_); 6.42 (s, 1H, CH_thiadiazole_), 4.00–3.85 (m, 6H, 2OCH_3_), ^13^CNMR (DMSO-*d*_*6*_*)*, *δ ppm*: 163.89 (C=O_amidic_), 151.47 (C_thiadiazole_), 147.75, 142.01, (2C_arom_.), 139.81, 131.65, 127.14, (3C_arom_.), 126.20, 121.47 (4CH_arom_.), 119.50, 112.39, 110.18 (5CH_arom_), 62.36, 61.73 (2OCH_3_) and 56.50 (CH_thiadiazole_).; C. F.: C_21_H_20_N_4_O_5_S_2_; M. W.: 472.53. Elemental Analysis: calc; C, 53.38; H, 4.27; N, 11.86; found; C, 53.40; H, 4.25; N, 11.90.

*N5,N5'-Bis(4-sulfamoylphenyl)-2,2′,3,3′-tetrahydro-[2,2′-bi(1,3,4-thiadiazole)]-5,5′-dicarboxamide ****(4)****:* Pale yellow solid, Yield (75%). Mp 0.260–262 °C. FT IR (KBr) ν max cm^−1^: 3375, 3297 (2NH, NH_2_), 3106 (CH-_arom._), 2972(CH-_aliph._), 1650 (C=O_amidic_, st), and 1152 (S=O, st). ^1^H-NMR (DMSO-d_6_), δ ppm: 10.45 (s, 1H, NH_amide_), 9.16 (s, 1H, NH_thiadiazole_), 7.91, 7.75 (m, 4H, CH_arom_.); 7.72 (s, 2H, NH_2_); 5.58 (s,1 H, CH_thiadiazole_), ^13^CNMR (DMSO-d_*6*_), *δ* ppm: 158.55 (C=O_amidic_), 141.64 (C_thiadiazole_), 139.02, 138.35 (2C_arom_.), 126.93, 120.26 (4CH_arom_.), 76.32 (CH_thiadiazole_).; C. F.: C_18_H_18_N_8_O_6_S_4_; M. W.: 570.63. Elemental Analysis: C, 37.89; H, 3.18; N, 19.64. Found; C, 37.91; H, 3.15; N, 19.58.

*N-(4-sulfamoylphenyl)-5-(3,4,5-trimethoxyphenyl)-4,5-dihydro-1,3,4-thiadiazole-2-carboxamide ****(5)****:* Pale yellow solid, Yield (75%). Mp 0.222–224 °C. FT IR (KBr) ν max cm^−1^: 3475, 3273, 3221 (2NH, NH_2_), 3105(CH-_arom._), 2993(CH-_aliph._), 1683 (C=O_amidic_, st), and 1160 (S=O, st). ^1^H-NMR (DMSO-d_6_), δ ppm: 10.49 (s, 1H, NH_amide_), 9.33 (s, 1H, NH_thiadiazole_), 7.96–7.23 (m, 6H, CH_arom._); 6.83 (s, 2H, NH_2_); 6.64 (s, 1H, CH_thiadiazole_), 3.86–3.32 (m, 9H, 3OCH_3_); ^13^CNMR (DMSO-d_6_), δ ppm: 161.52 (C=O_amidic_), 159.03 (C_thiadiazole_), 157.32, 153.64, 153.50 (3C-OCH_3_), 138.36, 137.33 (2C_arom_), 127.21 (2CH_arom_), 121.15, 120.22 (4CH_arom_), 75.07 (CH_thiadiazole_), 60.74 (OCH_3_) and 56.50 (2OCH_3_) ; C. F.: C_18_H_20_N_4_O_6_S_2_; M.W.: 452.50. Elemental Analysis: calc: C, 47.78; H, 4.46; N, 12.38; found: C, 47.80; H, 4.44; N, 12.35.

*5-Ethyl-N-(4-sulfamoylphenyl)-4,5-dihydro-1,3,4-thiadiazole-2-carboxamide ****(6)****:* Brownish yellow solid, Yield (71%). Mp .dec. > 300 °C. FT IR (KBr) ν max cm^-1^: 3475, 3456, 3381 (2NH, NH_2_), 3105 (CH-_arom._), 2993 (CH-_aliph._), 1677 (C=O_amidic_, st), and 1160 (S=O, st). ^1^H-NMR (DMSO-*d*_*6*_), *δ* ppm*:* 10.35 (s, 1H, NH_amide_), 9.28 (s, 1H, NH), 8.01–7.82 (m, 4H, CH_arom_); 7.29 (s, 2H, NH_2_), 6.03–5.93 (m, 1H, CH=CH_2_), 5.45–5.24 (dd, 2H, CH_2_), 4.08–4.06 (d, 1H, CH_thiadiazole_); ^13^CNMR (DMSO-*d*_*6*_), *δ* ppm*:* 165.13 (C=O_amidic_), 156.74 C_thiadiazole_), 141.02, 140.22 (2C_arom_), 132.51 (CH–CH_2_), 127.03, 120.99 (4CH_arom_), 106.20, 104.21 (CH_2_) and 56.78 (CH_thiadiazole_). C. F.: C_11_H_12_N_4_O_3_S_2_; M.W.: 312.36. Elemental Analysis: calc: C, 42.30; H, 3.87; N, 17.94; found: C, 42.33; H, 3.88; N, 17.88.

*5-(4-Hydroxy-3-methoxyphenyl)-N-(4-sulfamoylphenyl)-4,5-dihydro-1,3,4-thiadiazole-2-carboxamide ****(7):*** White solid, Yield (88%). Mp 0.256–258 °C. FT IR (KBr) ν max cm^−1^: 3495 (OH, st), 3435, 3394, 3280 (NH, NH_2_), 3057 (CH-_arom._) 2975(CH-_aliph_.), 1693 (C = O_amidic_, st), and 1157 (S=O, st). ^1^H-NMR (DMSO-d_6_), δ ppm: 13.68 (s, 1H, OH), 10.45 (s, 1H, NH_amide_), 9.25 (s, 1H, NH_thiadiazole_), 7.92–7.76 (m, 4H, CH_arom_), 7.39–7.22 (m, 3H, CH_arom_); 6.90–6.79 (d, 2H, NH_2_); 6.60 (s, 1H, CH_thiadiazole_), 3.78 (s, 3H, OCH_3_); ^13^CNMR (DMSO-d_6_), δ ppm: 164.45 (C=O_amidic_), 158.91 (C_thiadiazole_), 151.24, 148.70 (2C_arom_), 141.81, 141.12 (2C_arom_), 140.24 (C_arom_), 127.23, 121.08 (4CH_arom_), 120.19, 116.12, 111.84 (3CH_arom_), 74.94 (CH_thiadiazole_) and 55.54 (OCH_3_); C. F.: C_16_H_16_N_4_O_5_S_2_; M. W.: 408.45. Elemental Analysis: calc: C, 47.05; H, 3.95; N, 13.72; found: C, 47.11; H, 3.91; N, 13.69.

*5-(4-Chlorophenyl)-N-(4-sulfamoylphenyl)-4,5-dihydro-1,3,4-thiadiazole-2-carboxamide ****(8)***: Yellow solid, Yield (75%). Mp 0.260–262 °C. FT IR (KBr) ν max cm^−1^: 3340, 3231 (NH, NH_2_), 3057 (CH_-arom_.) 2975 (CH-_aliph_.), 1671 (C=O_amidic_,st), and 1159 (S = O, st). ^1^H-NMR (DMSO-d_6_), δ ppm: 10.50 (s, 1H, NH_amide_), 9.41 (s, 1H, NH_thiadiazole_), 7.91–7.48 (m, 8H, CH_arom_); 7.23 (s, 2H, NH_2_); 6.68 (s, 1H, CH_thiadiazole_); ^13^CNMR (DMSO-d_6_), δ ppm: 171.44 (C=O_amidic_), 165.69 (C_thiadiazole_), 158.73, 156.41 (2C_arom_), 140.37, 137.39 (2C_arom_), 130.45, 126.93 (4CH_arom_),, 121.12, 120.23 (4CH_arom_), and 73.67 (CH_thiadiazole_); C. F.: C_15_H_13_ClN_4_O_3_S_2_; M. W.: 396.86. Elemental Analysis: calc: C, 45.40; H, 3.30; Cl, 8.93; N, 14.12; calc: C, 45.45; H, 3.28; Cl, 8.90; N, 14.10.

*5-(Furan-2-yl)-N-(4-sulfamoylphenyl)-4,5-dihydro-1,3,4-thiadiazole-2-carboxamide ****(9)***: Greenish yellow solid, Yield (85%). Mp 0.250–252 °C. FT IR (KBr) ν max cm^-1^: 3454, 3349, 3249 (NH, NH_2_), 3060 (CH-_arom_.) 2927 (CH-_aliph._), 1668 (C=O_amide_,st), and 1157 (S=O, st). ^1^H-NMR (DMSO-*d*_*6*_), δ ppm: 10.49 (s, 1H, NH amide), 9.34 (s, 1H, NH_thiadiazole_), 7.92–7.23 (m, 7H, CH_arom_.); 6.70 (s,1H, CH_thiadiazole_); 6.46 (s, 2H, NH_2_); ^13^CNMR (DMSO-*d*_*6*_), δ ppm: 164.30 (C=O_amidic_), 156.89 (C_thiadiazole_), 153.43 (C_furfural_), 147.77, 140.30 (2C_arom_), 137.88 (CH_furfural_), 126.94, 121.15 (4CH_arom_), 120.31, 115.09 (2CH_furfural_), and 67.14 (CH_thiadiazole_); C. F.: C_13_H_12_N_4_O_4_S_2_; M. W.: 352.38. Elemental Analysis: calc: C, 44.31; H, 3.43; N, 15.90; found: C, 44.35; H, 3.41; N, 15.88.

*5-(2-Hydroxyphenyl)-N-(4-sulfamoylphenyl)-4,5-dihydro-1,3,4-thiadiazole-2-carboxamide (****10)***: Yellow solid, Yield (75%). Mp 0.288–290 °C. FT IR (KBr) ν max cm^−1^: 3451 (OH, st), 3351, 3263, 3168 (NH, NH_2_), 3025 (CH-_arom_.) 2934(CH-_aliph_.), 1687(C=O_amide_, st), and 1161 (S=O, st). ^1^H-NMR (DMSO-d_6_), δ ppm: 10.44 (s, 1H, NH_amide_), 9.93 (s, 1H, OH), 9.20 (s, 1H, NH_thiadiazole_), 7.93–7.18 (m, 8H, CH_arom_.); 6.87 (s, 2H, NH_2_); 6.72 (s,1H, CH_thiadiazole_); ^13^CNMR (DMSO-d_6_), δ ppm: 166.48 (C=O_amidic_), 165.04 (C_thiadiazole_), 157.90, 155.69 (2C_arom_), 141.23, 140.13 (2C_arom_), 133.70, 130.12, 127.03 (3CH_arom_),, 120.34, 116.99 (4CH_arom_), and 69.38 (CH_thiadiazole_), C. F.: C_15_H_14_N_4_O_4_S_2_; M. W.: 378.42. Elemental Analysis: calc. C, 47.61; H, 3.73; N, 14.81; found: C, 47.63; H, 3.71; N, 14.79.

*5-(4-methoxyphenyl)-N-(4-sulfamoylphenyl)-4,5-dihydro-1,3,4-thiadiazole-2-carboxamide ****(11)***: White solid, Yield (86%). Mp 0.288–286 °C. FT IR (KBr) ν max cm^−1^: 3350, 3247, 3163 (NH, NH_2_), 3057 (CH-_arom_.) 2975 (CH-_aliph._), 1676 (C=O_amide_,st), and 1160 (S=O, st). ^1^H-NMR (DMSO-d_6_), δ ppm: 10.46 (s, 1H, NH_amide_), 9.30 (s, 1H, NH_thiadiazole_), 7.92–7.23 (m, 8H, CH_arom_.); 6.98 (s, 2H, NH_2_); 6.63 (s, 1H, CH_thiadiazole_). 3.77 (s, 3H, OCH_3_); ^13^CNMR (DMSO-d_6_), δ ppm: 172.41 (C=O_amide_), 164.45 (C_thiadiazole_), 162.87, 159.09 (2C_arom_), 157.31, 141.29 (2C_arom_), 130.36, 127.28 (4CH_arom_), 121.10, 116.10 (4CH_arom_), 74.68 (CH_thiadiazole_) and 55.86 (OCH_3_); C. F.: C_16_H_16_N_4_O_4_S_2_; M. W.: 392.45. Elemental Analysis: calc.: C, 48.97; H, 4.11; N, 14.28; found: C, 48.00; H, 4.08; N, 14.26.

*N-(4-Sulfamoylphenyl)-1,3,4-thiadiazole-2-carboxamide (****12)***: White solid, Yield (85%). Mp 0.234–236 °C. FT IR (KBr) ν max cm^-1^; 3347, 3269, 3182 (NH, NH_2_), 3030 (CH_-arom_.) 2936(CH-_aliph._), 1681 (C=O_amide_, st), and 1155 (S=O, st). ^1^H-NMR (DMSO-d_6_), δ ppm: 10.47 (s, 1H, NH_amide_), 8.80 (s, 1H, =CH_thiadiazole_), 7.95–7.28 (m, 4H, CH_arom_.); ^13^CNMR (DMSO-d_6_), δ ppm: 167.92 (C=O_amide_), 158.96, 153.61 (2C_thiadiazole_), 140.80, 140.11 (2C_arom_), 127.08, 120.49 (4CH_arom_); C. F.: C_9_H_8_N_4_O_3_S_2_; M. W.: 284.31. Elemental Analysis: C, 38.02; H, 2.84; N, 19.71 found: C, 38.12; H, 2.80; N, 19.69.

## Conclusion

In this paper, we are described about the importance of developing a new insecticidal agent and designing of a new and novel chemical compound with low toxicity and positive environmental impact. we are developed a new and novel sulfonamide based dihydro thiadiazoles to address the problems associated with existing chemical pesticides such as impact on environment, health rick in both humans and animals and insecticidal resistance. To address the all issues, we are focused on sulfonamide based dihydro thiadiazoles, synthesized, characterized well with spectral data and elemental analysis. Based on our data, toxic activity of new sulfonamide hybrid with thiadiazole derivatives containing chlorophenyl sulfonamide-thiadiazole that compound **8** is more effective against fourth and second of *S. littoralis* larvae than the other sulfonamide-thiadiazole synthesized compounds. Evaluation of the latent effects of the studied components on various biological parameters, such as adult longevity, pupal weight, proportion of normal, deformed pupae and adult emergency, fecundity and egg hatchability, were also carried out in an effort to slightly improve insecticidal compounds. The chlorophenyl, sulfonamide, and thiadiazole moiety which are presence in the chemical structure of component **8** may be the source of its high level of efficacy. In accordance with the computed LC_50_ values of sulfonate containing the thiadiazole moiety, **8**, **10**, **3**, **7**, and **11** showed the most potent toxic effect, with LC_50_ values of 27.65, 29.01, 29.60, 30.06, and 31.02 ppm, respectively. When we looked at the activity line as the following order: **8 > 10 > 3 > 7 > 11 > 5 > 9 > 2 > 4 > 6 > 12,** which suggested that the treated strain of *S. littoralis* had a homologous response and had a variety of responses to the target synthesized products.

### Supplementary Information

Supplementary Figures.

## Data Availability

All information generated or examined during this inquiry is contained in this published article and it’s supporting information files.
