# Supplementary material for: Green synthesis, biological and molecular docking of some novel sulfonamide thiadiazole derivatives as potential insecticidal against Spodoptera littoralis
Source: Sci Rep. 2023 Nov 6;13:19142. doi: 10.1038/s41598-023-46602-1 (PMC10628220; doi:10.1038/s41598-023-46602-1)
Supplement: Supplementary file 1 — Supplementary Figures. [file 41598_2023_46602_MOESM1_ESM.docx]

**Green Synthesis, Biological and Molecular Docking of some Novel Sulfonamide Thiadiazole Derivatives as Potential Insecticidal against *Spodoptera littoralis***

**Ahmed M. El-Saghier^1*^, Souhaila S. Enaili^1,2^, Asmaa M. Kadry^1^, Aly Abdou^1^, Mohamed A. Gad^3^**

**^1^Chemistry Department, Faculty of Science, Sohag University, 282524 Sohag, Egypt**

**^2^Chemistry Department, Faculty of Science, Al Zawiya University, Al Zawiya, Libya**

**^3^Research Institute of Plant Protection, Agricultural Research Center, 12619 Giza, Egypt**


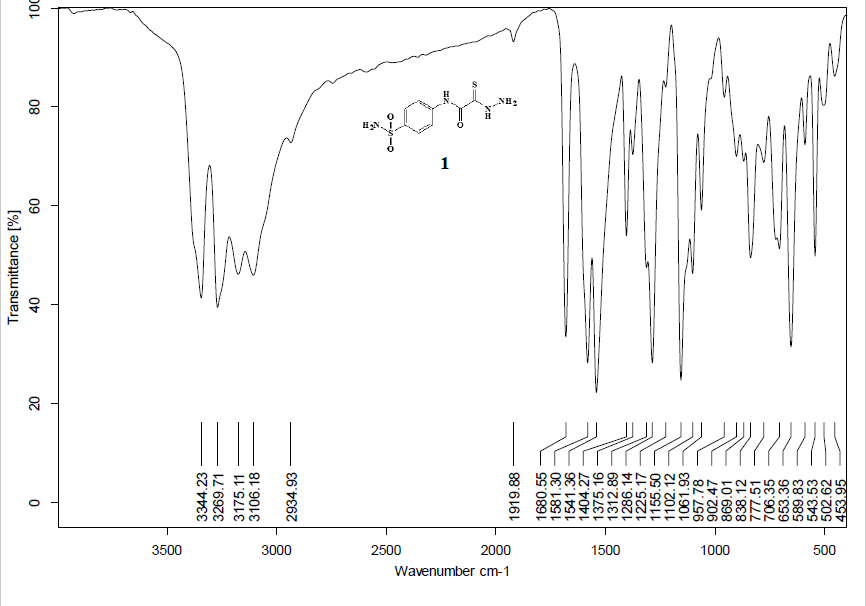


**Figure S1**: IR Spectrum of compound **1**


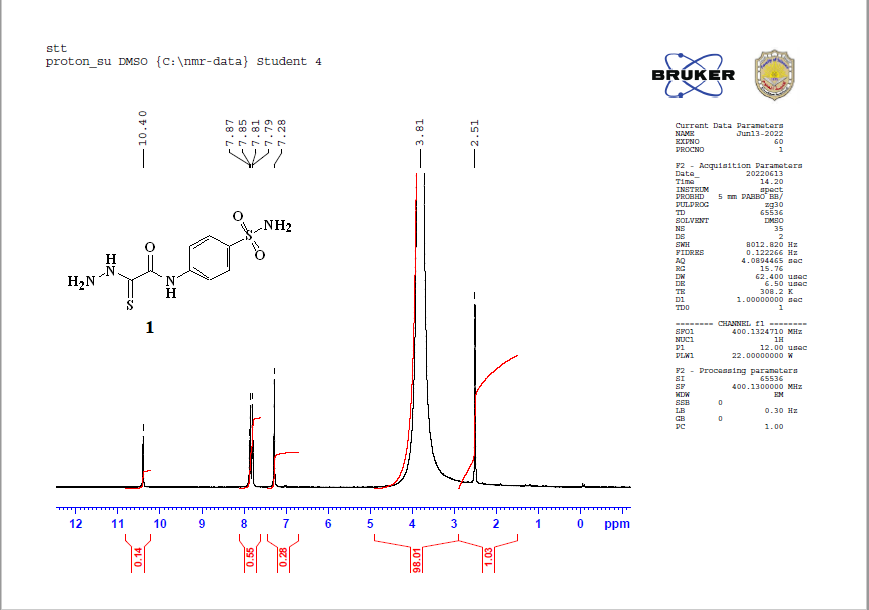


**Figure S2**: ^1^HNMR Spectrum of compound **1**


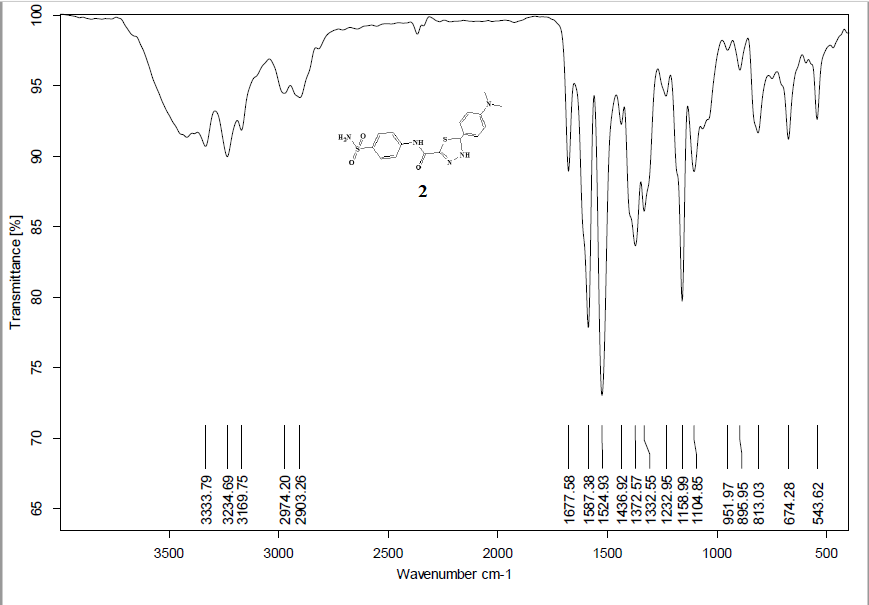


**Figure S3**: IR Spectrum of compound **2**


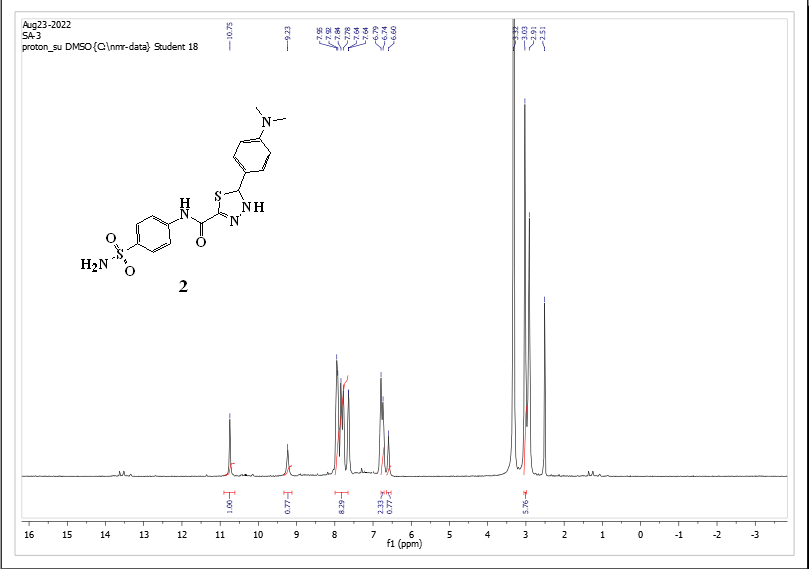


**Figure S4**: ^1^HNMR Spectrum of compound **2**


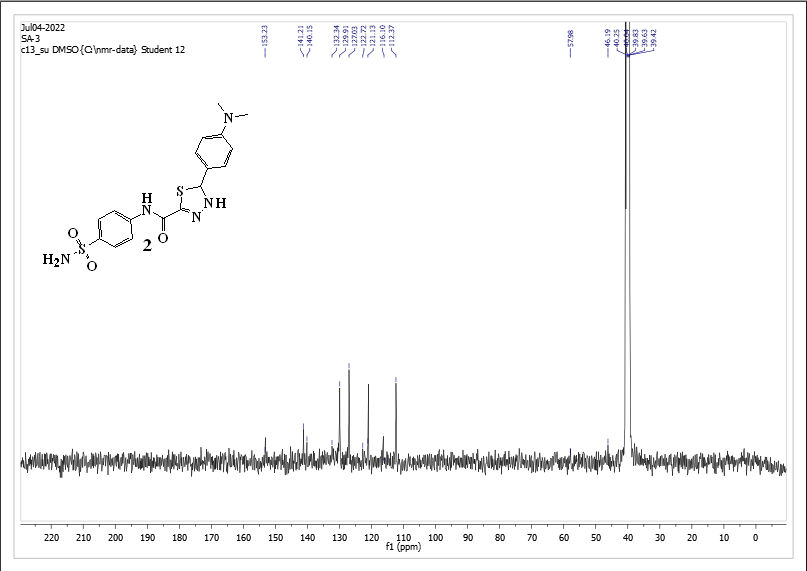


**Figure S5**: ^13^CNMR Spectrum of compound **2**


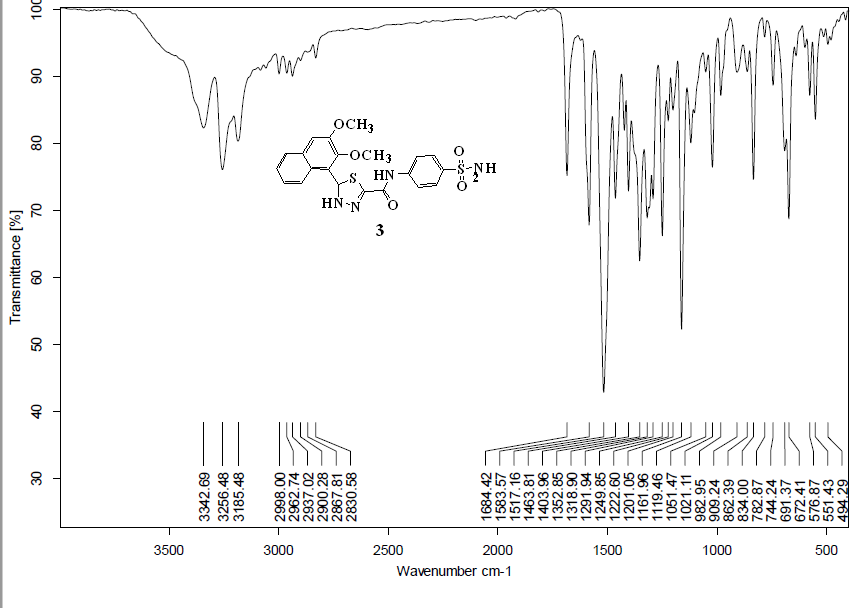


**Figure S6**: IR Spectrum of compound **3**


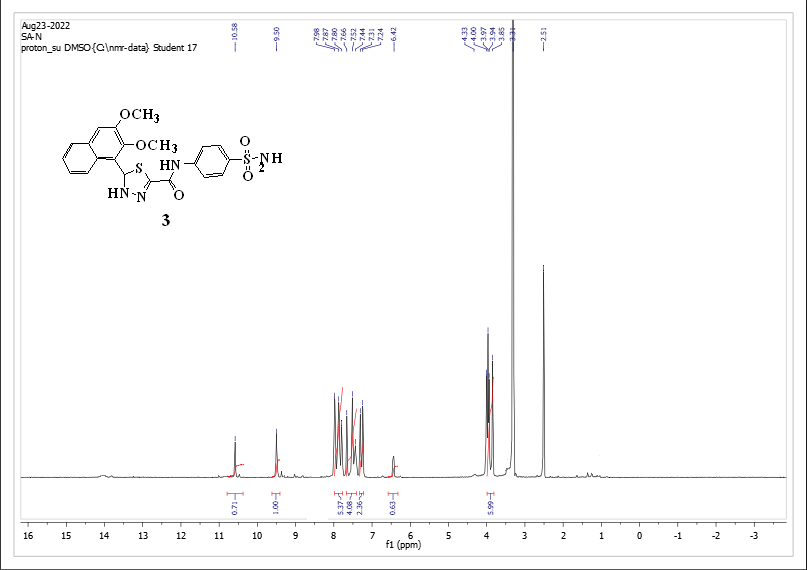


**Figure S7**: ^1^HNMR Spectrum of compound **3**


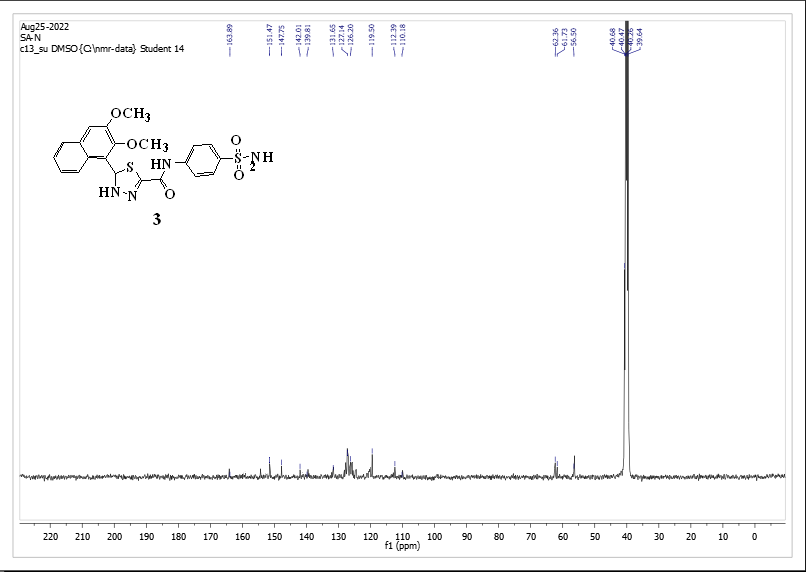


**Figure S8**: ^13^CNMR Spectrum of compound **3**


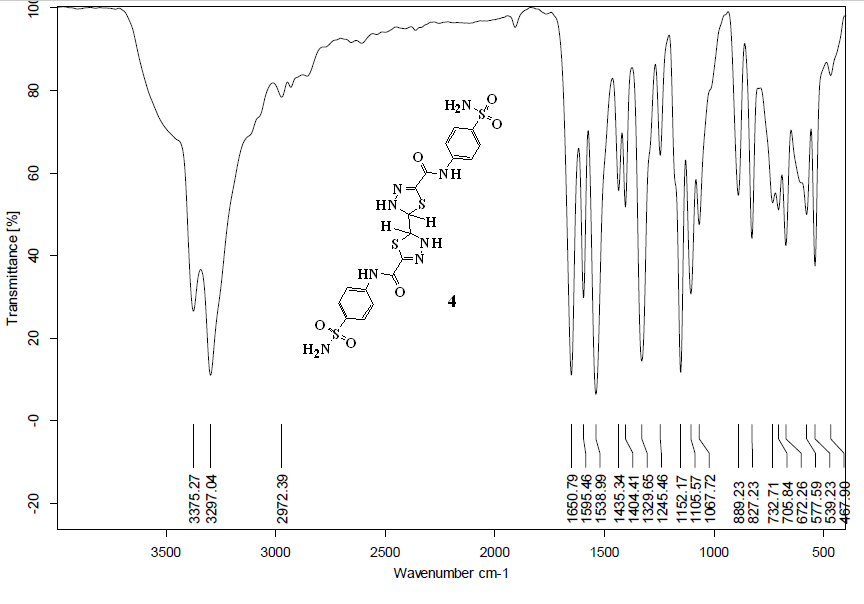


**Figure S9**: IR Spectrum of compound **4**


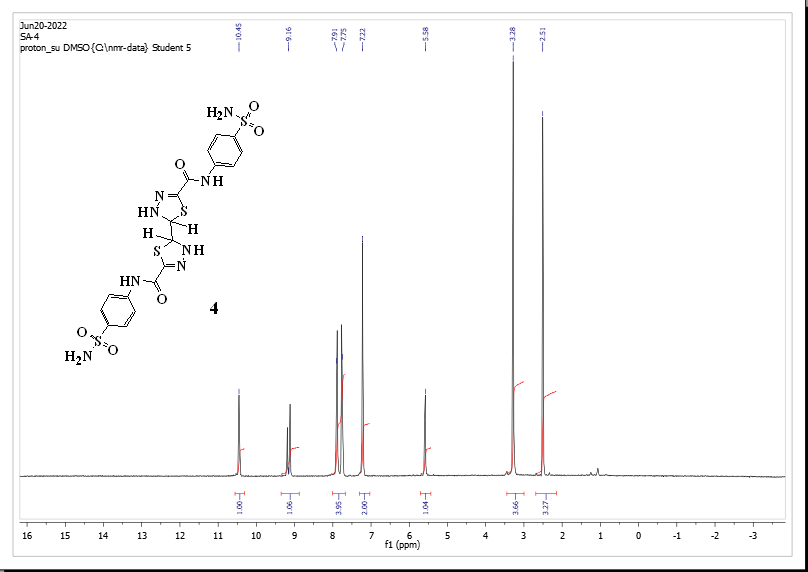


**Figure S10**: ^1^HNMR Spectrum of compound **4**


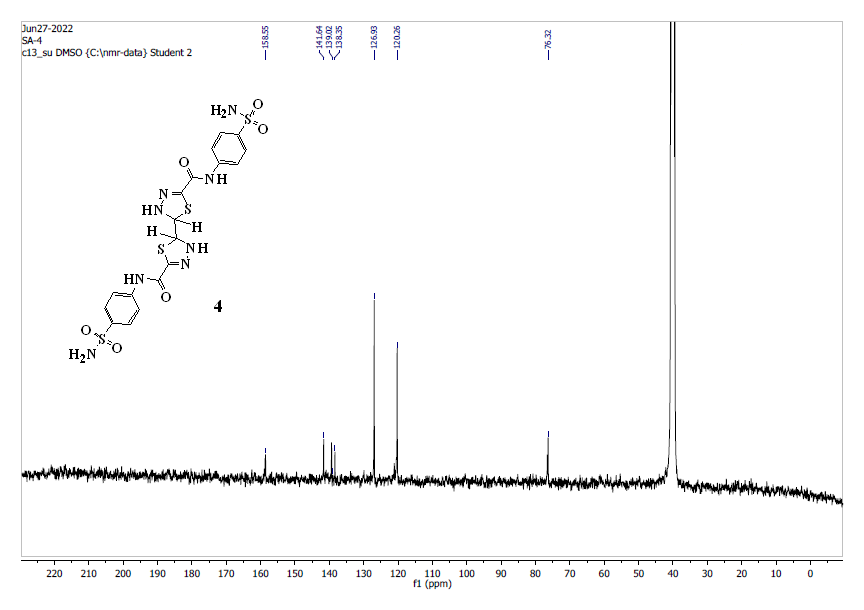


**Figure S11**: ^13^C-NMR Spectrum of compound **4**


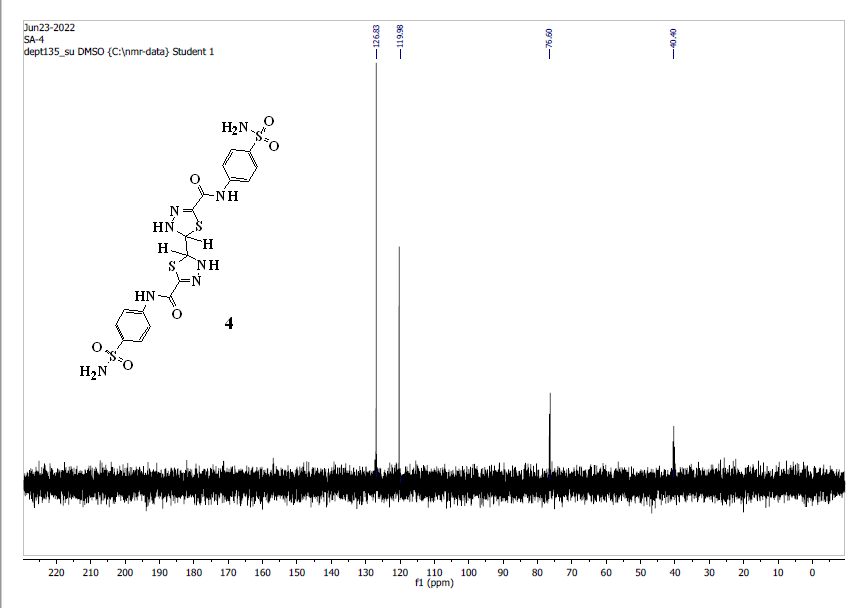


**Figure S12**: DEPT Spectrum of compound **4**


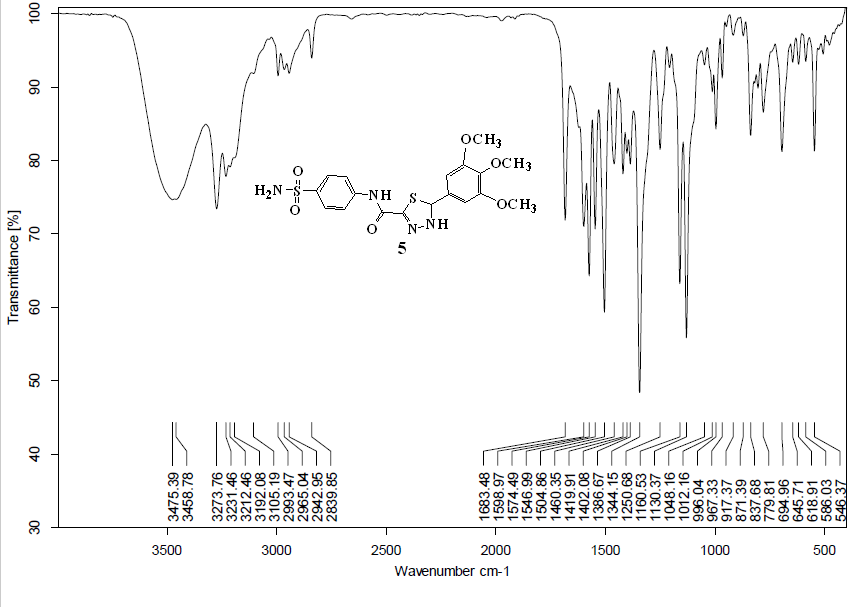


**Figure S13**: IR Spectrum of compound **5**


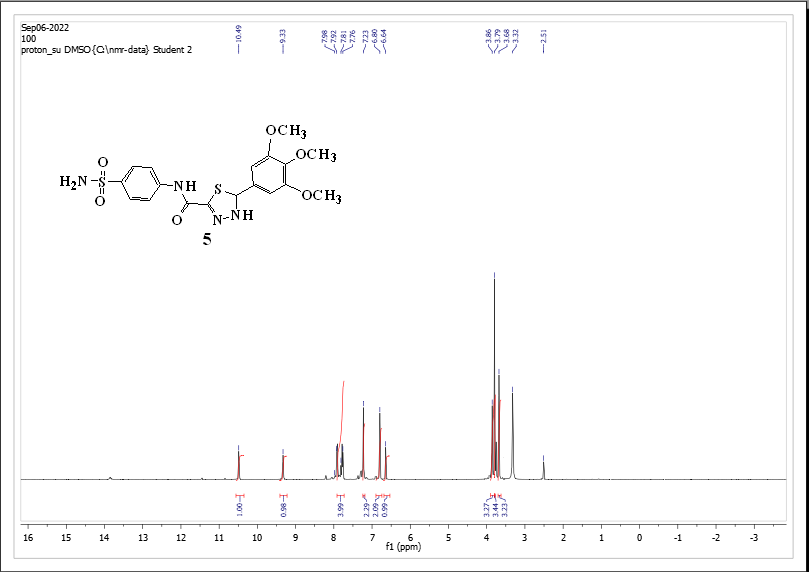


**Figure S14**: ^1^HNMR Spectrum of compound **5**


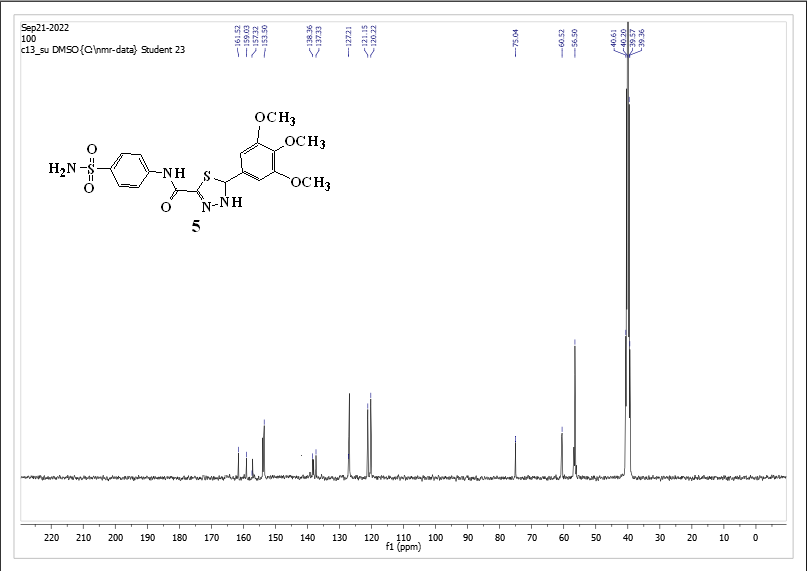


**Figure S15**: ^13^CNMR Spectrum of compound **5**


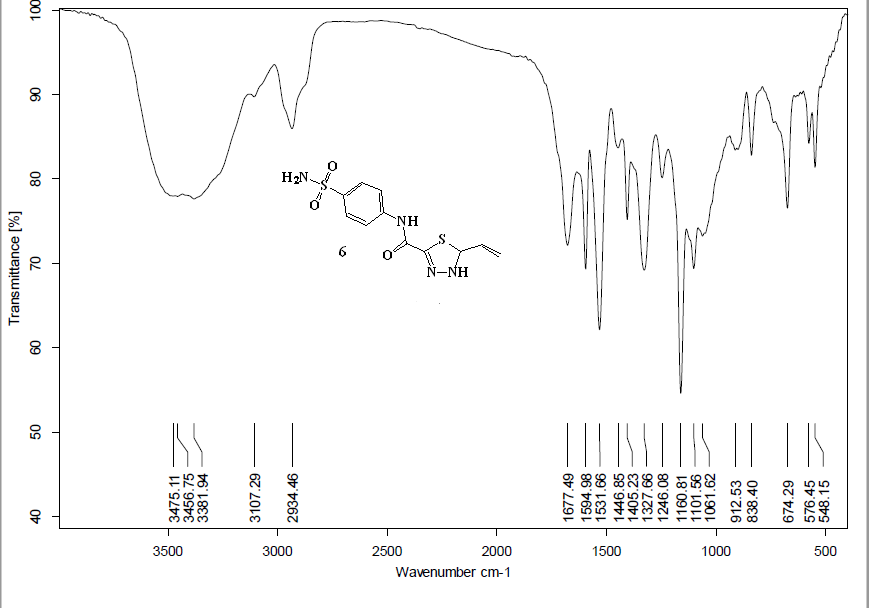


**Figure S16**: IR Spectrum of compound **6**


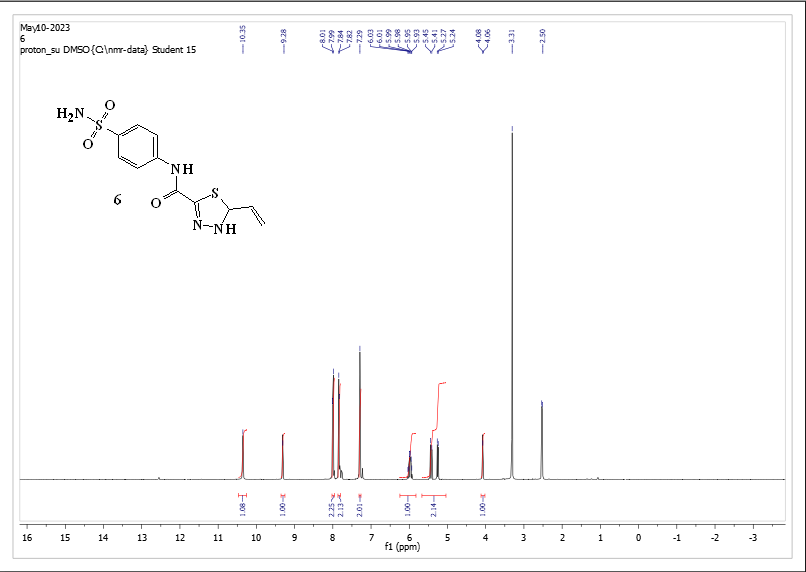


**Figure S17**: ^1^HNMR Spectrum of compound **6**


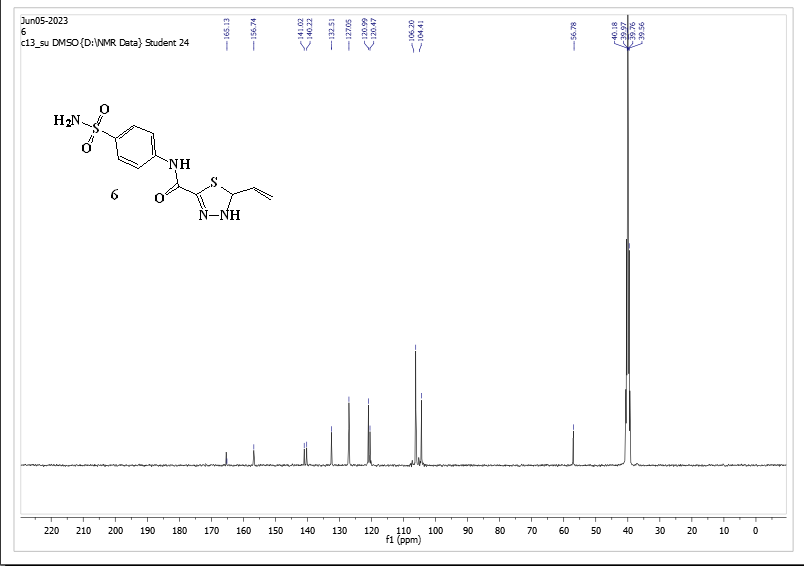


**Figure S18**: ^13^CNMR Spectrum of compound **6**


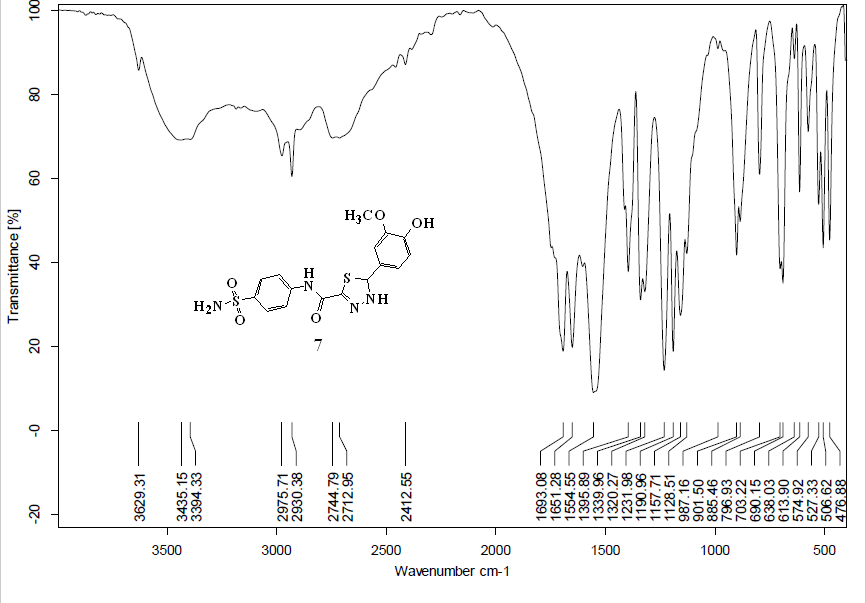


**Figure S19**: IR Spectrum of compound **7**


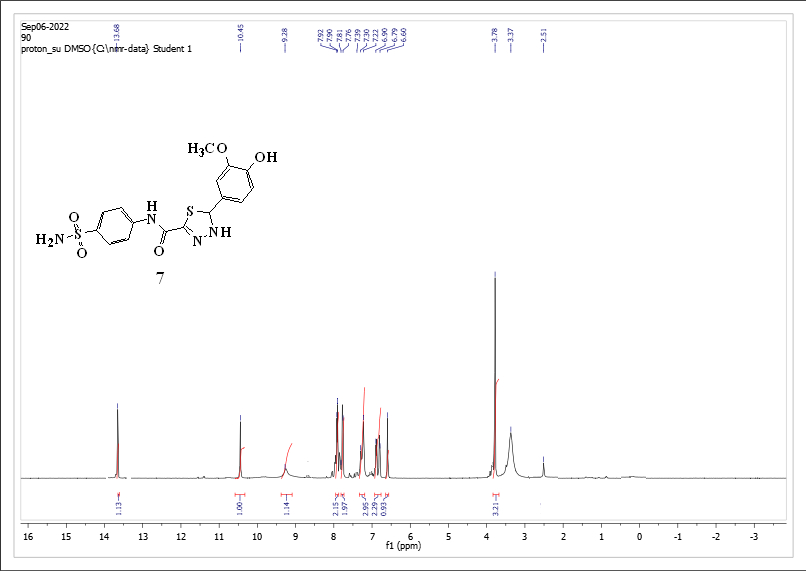


**Figure S20**: ^1^HNMR Spectrum of compound **7**


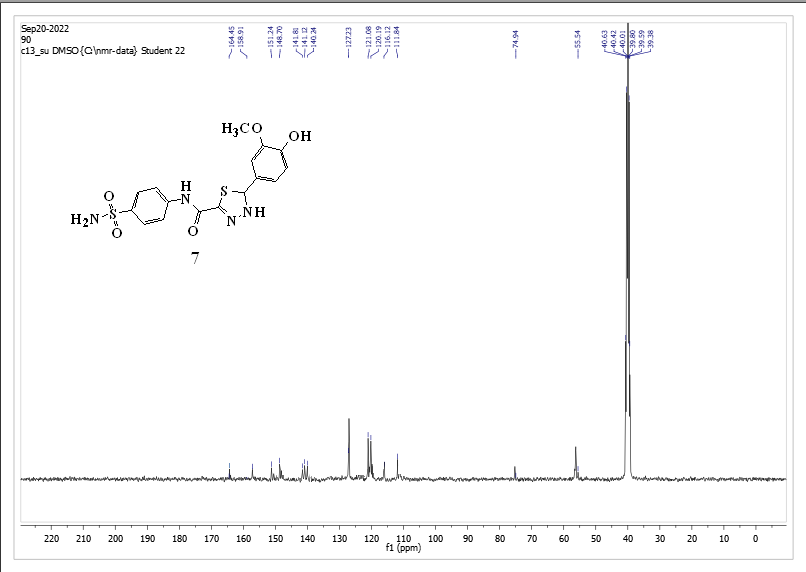


**Figure S21**: ^13^CNMR Spectrum of compound **7**


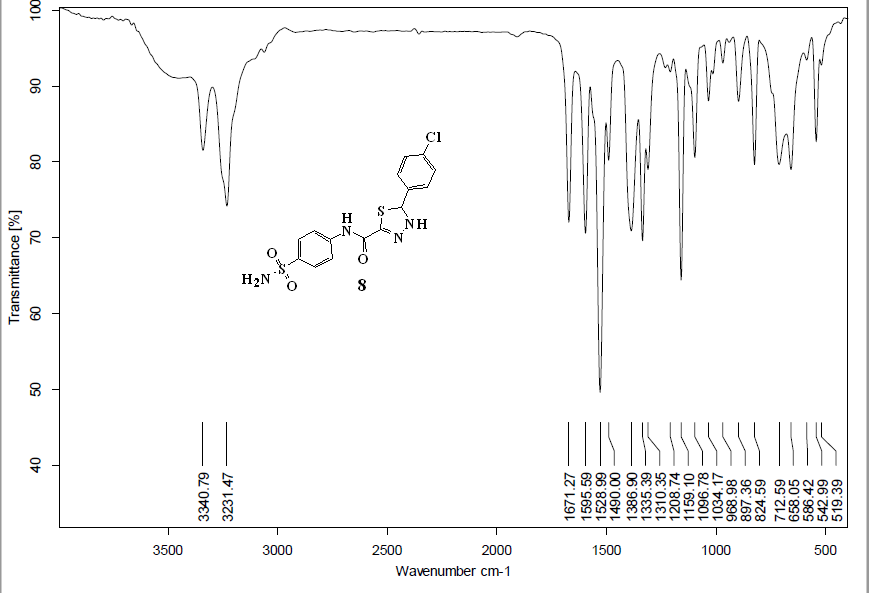


**Figure S22**: IR Spectrum of compound **8**


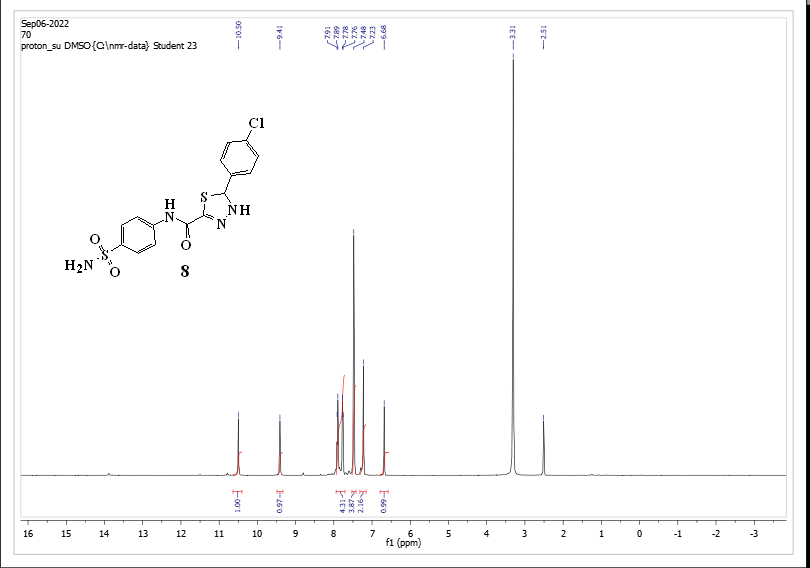


**Figure S23**: ^1^HNMR Spectrum of compound **8**


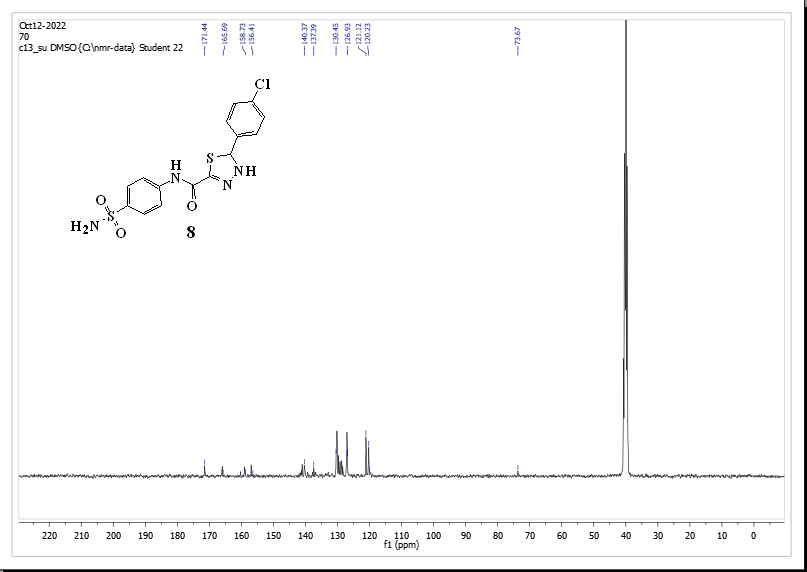


**Figure S24**: ^13^CNMR Spectrum of compound **8**


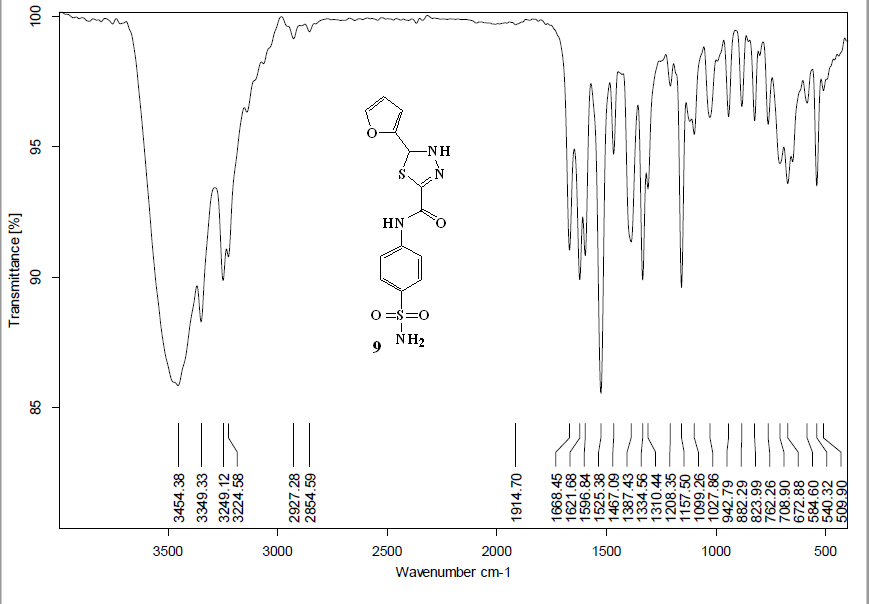


**Figure S25**: IR Spectrum of compound **9**


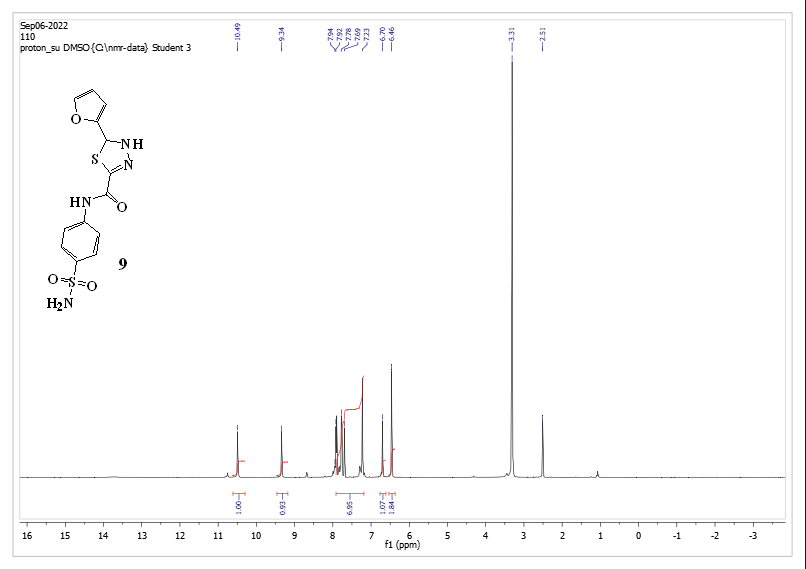


**Figure S26**: ^1^HNMR Spectrum of compound **9**


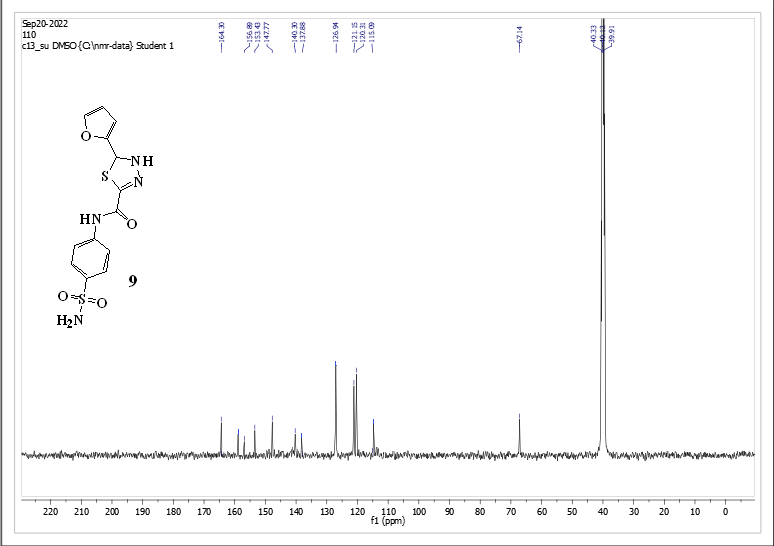


**Figure S27**: ^13^CNMR Spectrum of compound **9**


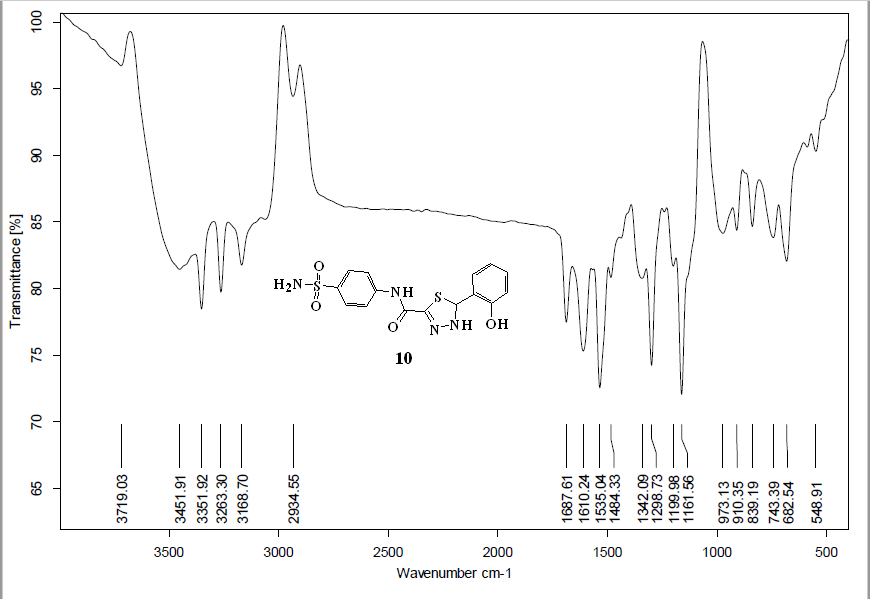


**Figure S28**: IR Spectrum of compound **10**


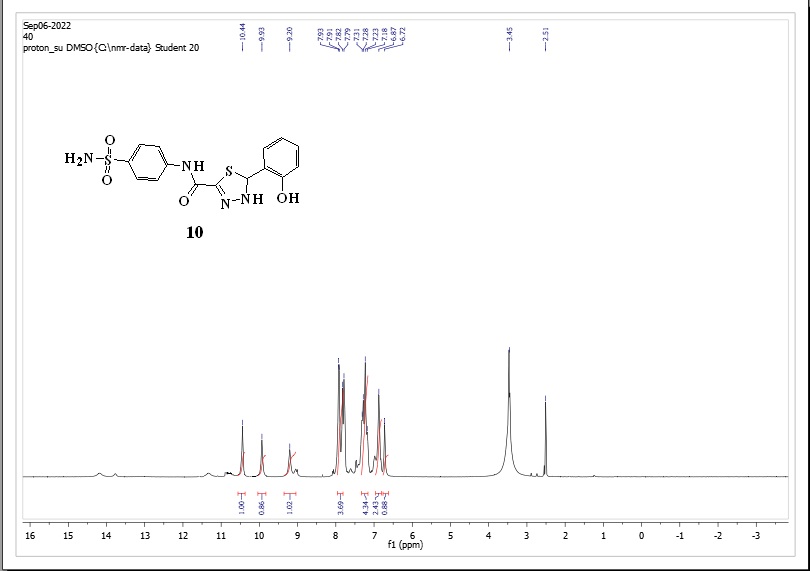


**Figure S29**: ^1^HNMR Spectrum of compound **10**


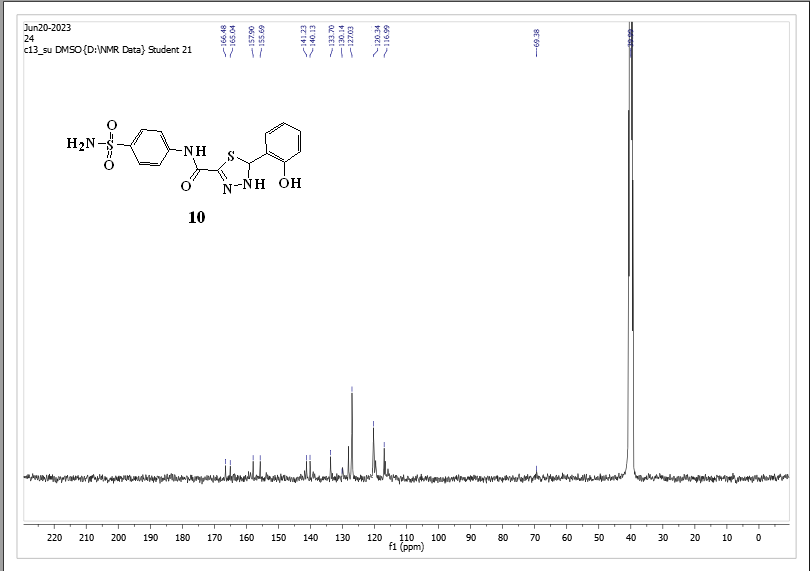


**Figure S30**: ^13^CNMR Spectrum of compound **10**


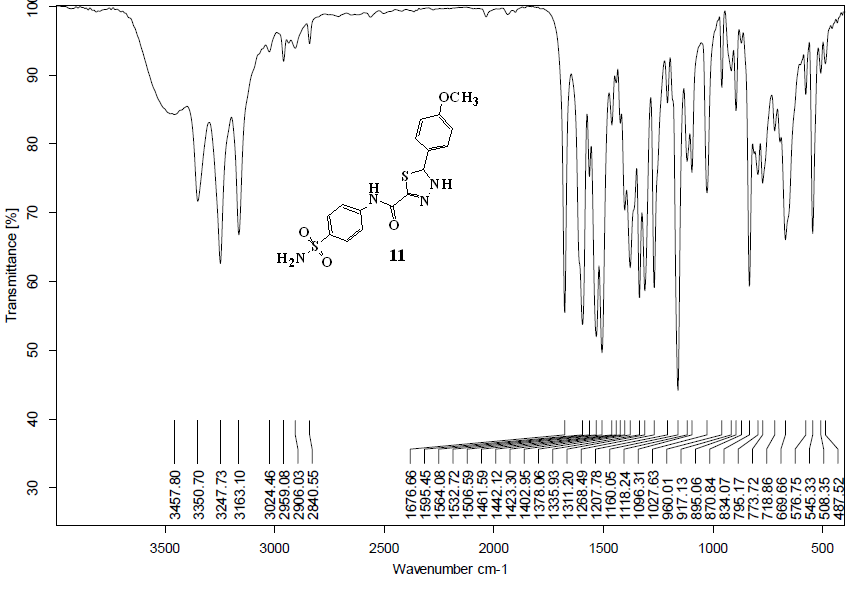


**Figure S31**: IR Spectrum of compound **11**


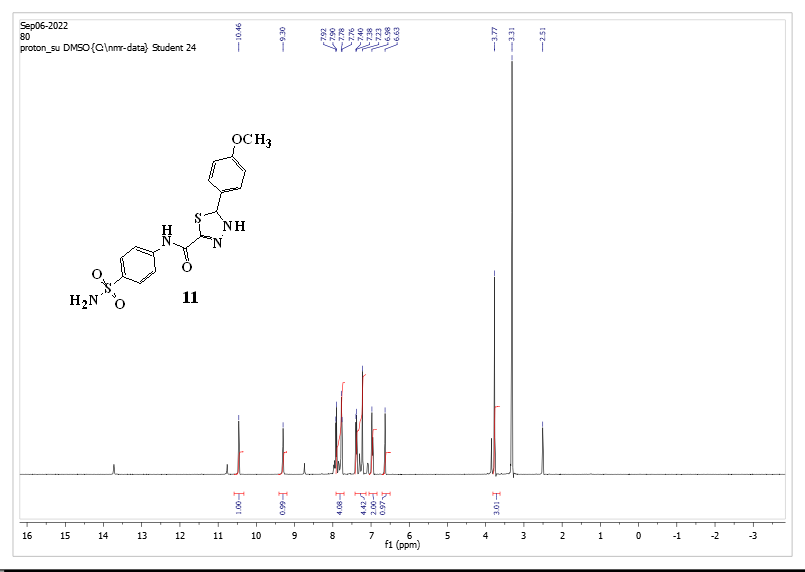


**Figure S32**: ^1^HNMR Spectrum of compound **11**


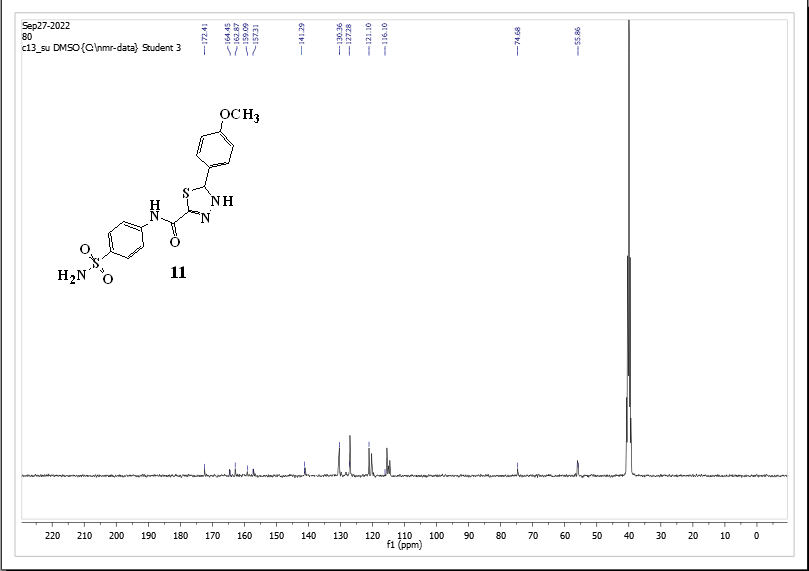


**Figure S33**: ^13^CNMR Spectrum of compound **11**


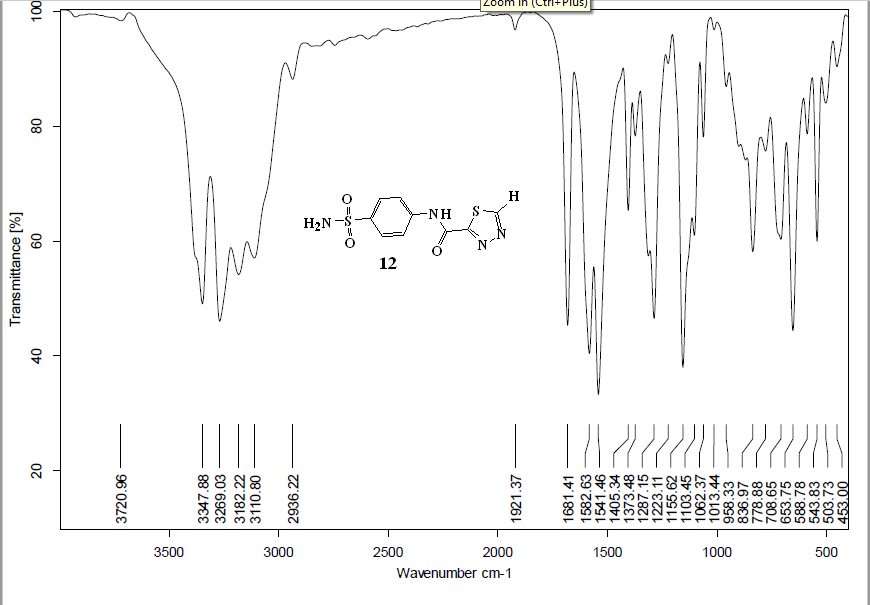


**Figure S34**: IR Spectrum of compound **12**


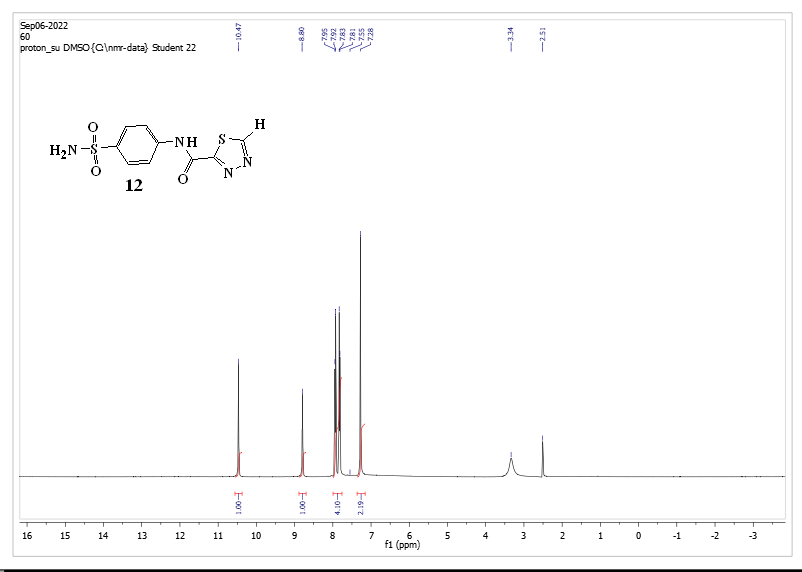


**Figure S35**: ^1^HNMR Spectrum of compound **12**


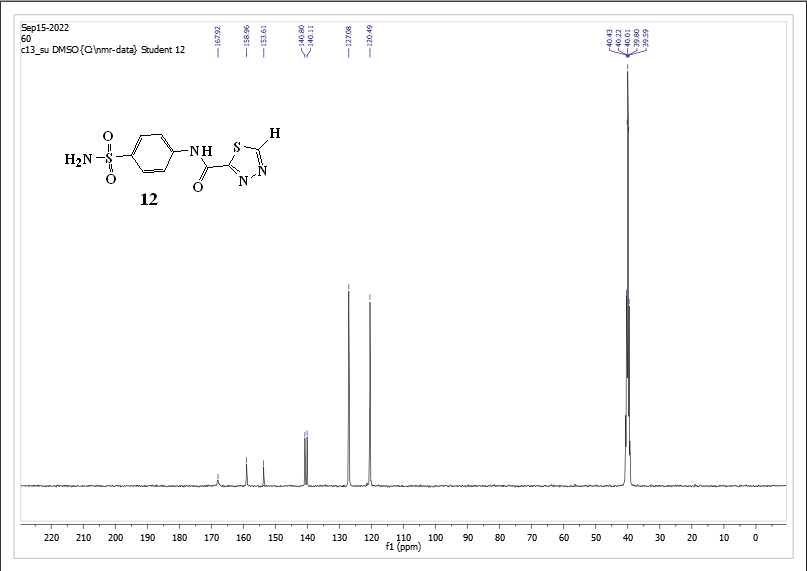


**Figure S36**: ^13^CNMR Spectrum of compound **12**

| 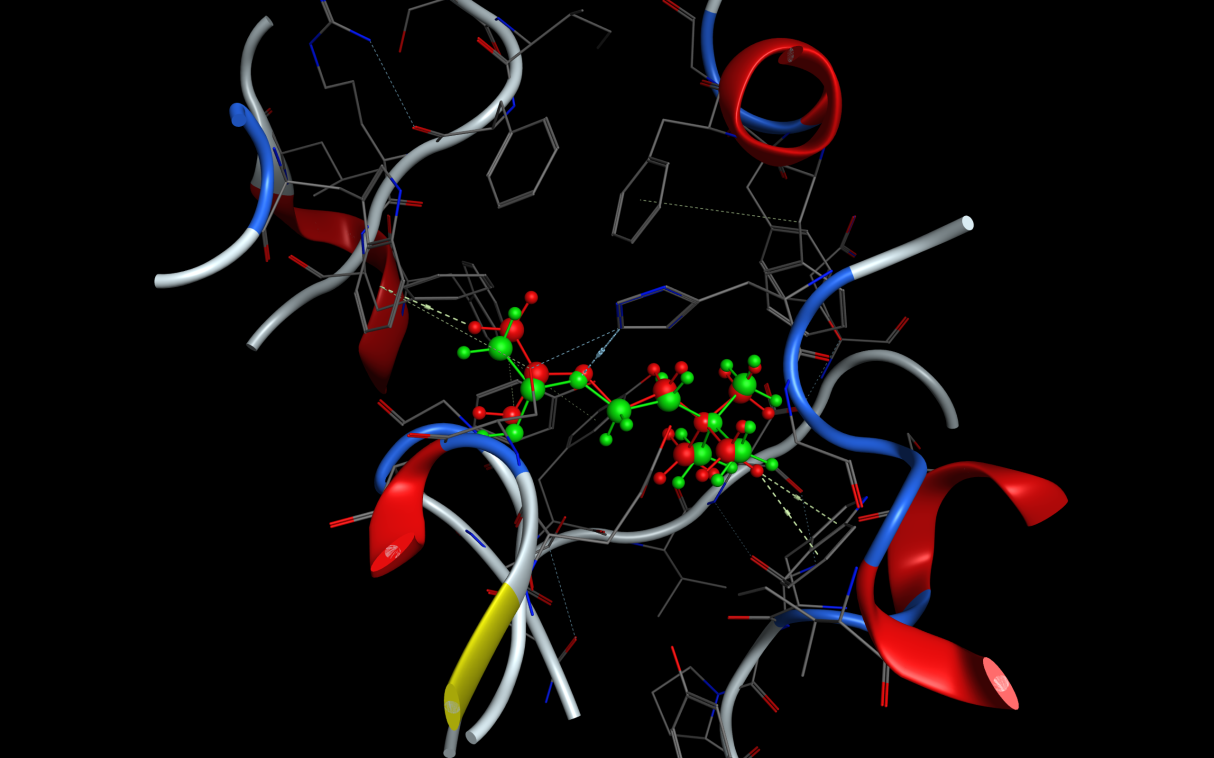 |
| --- |

**Figure S37**: 3D representation of the superimposition of the co-crystallized (red) and the re-docking pose (green) of the native ligand.

| **2** | **3** |
| --- | --- |
| 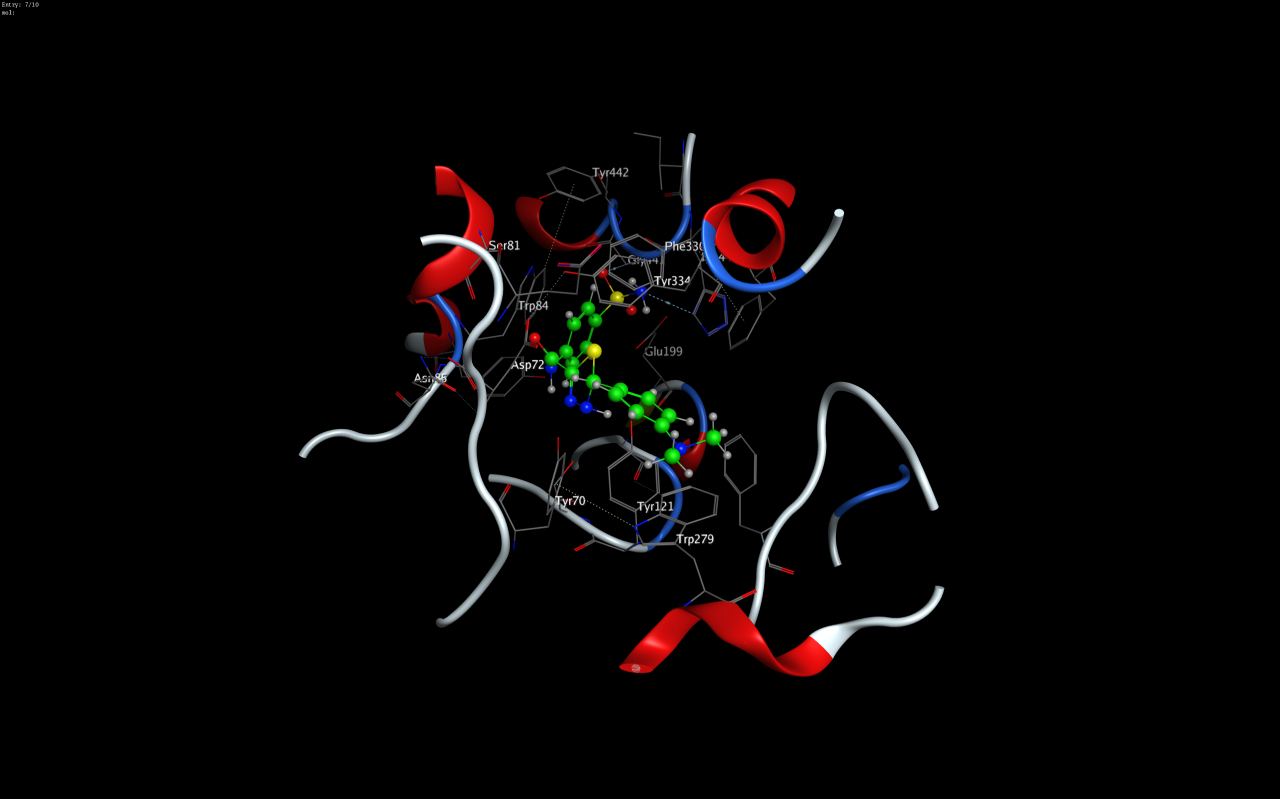 | 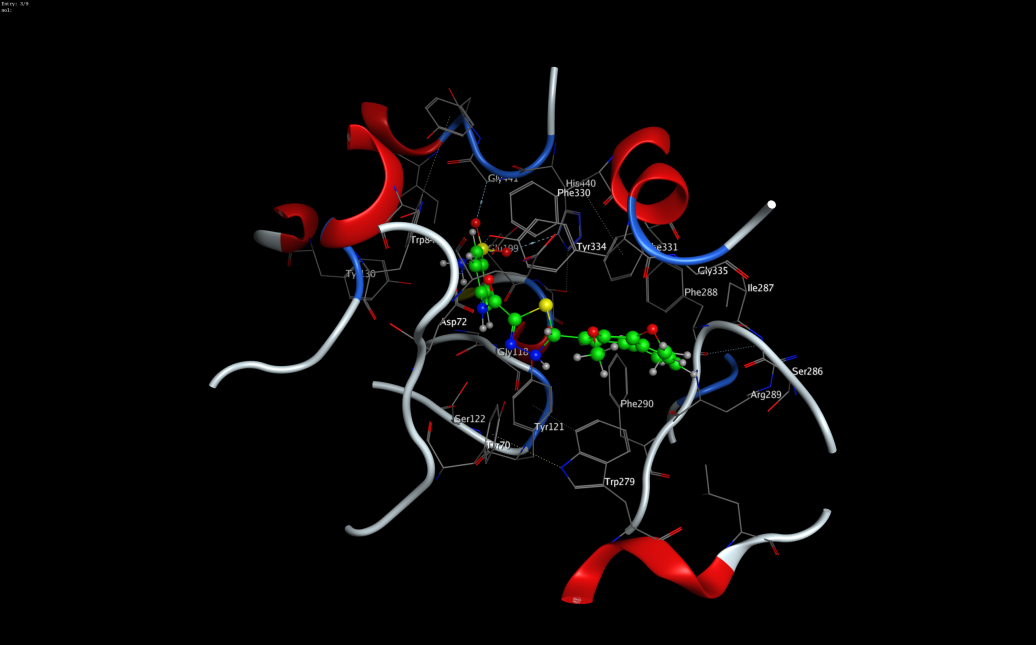 |
| **4** | **5** |
| 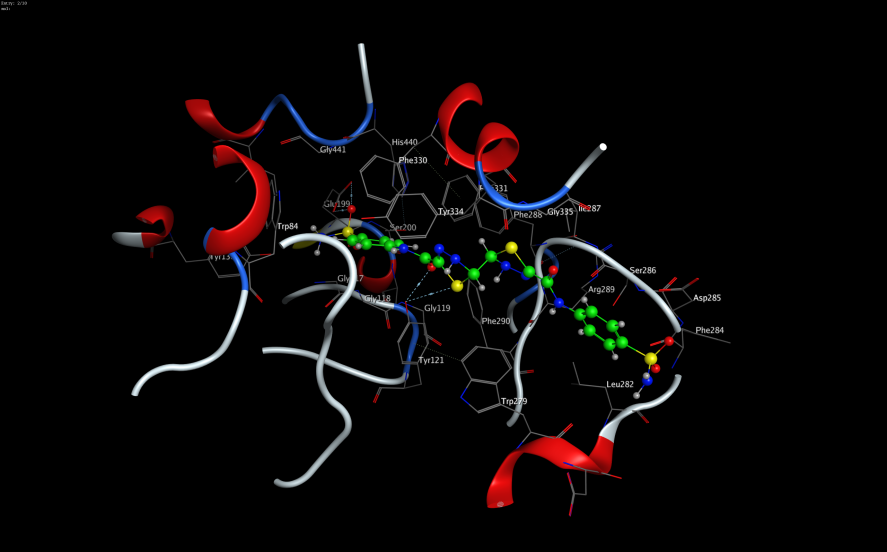 | 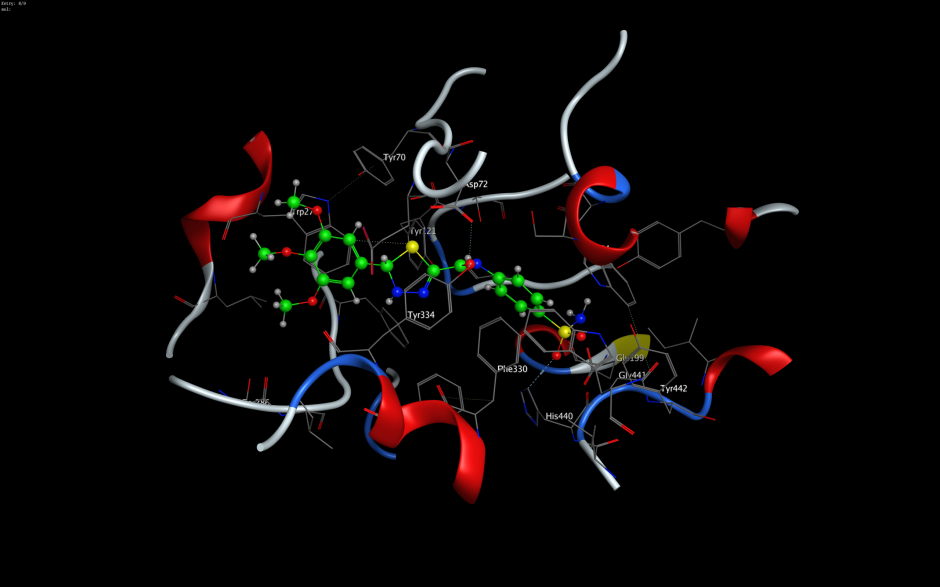 |
| **6** | **7** |
| 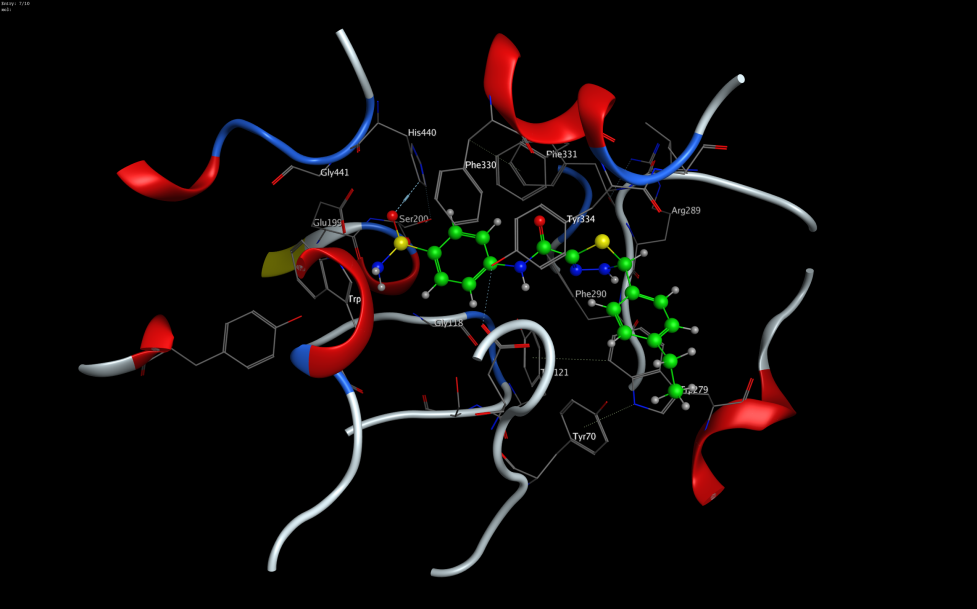 | 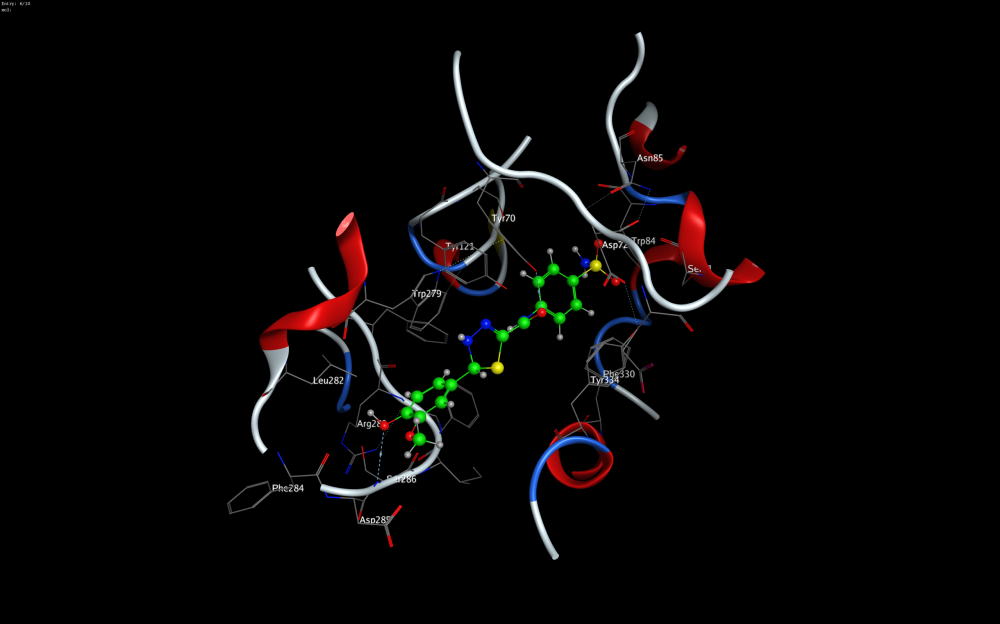 |
| **8** | **9** |
| 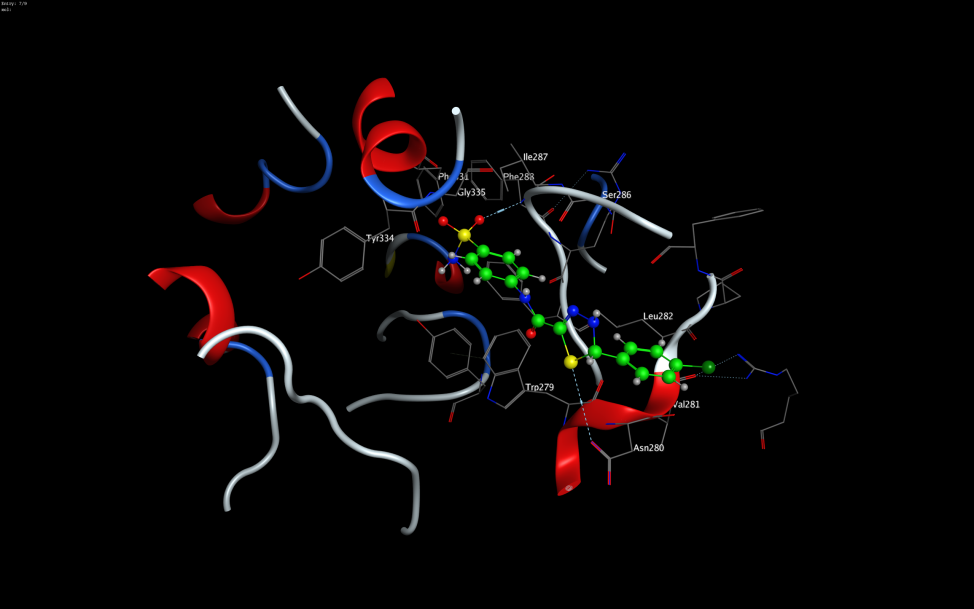 | 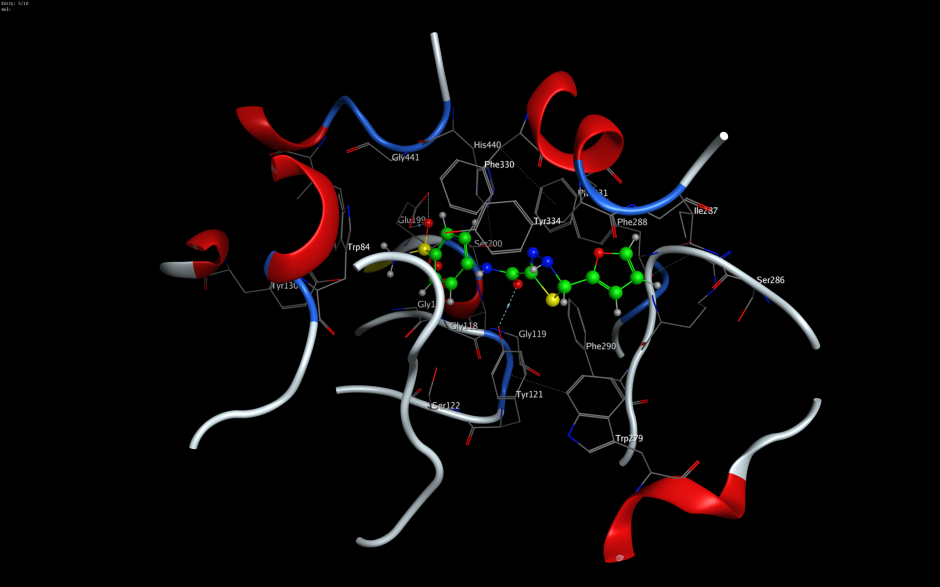 |
| **10** | **11** |
| 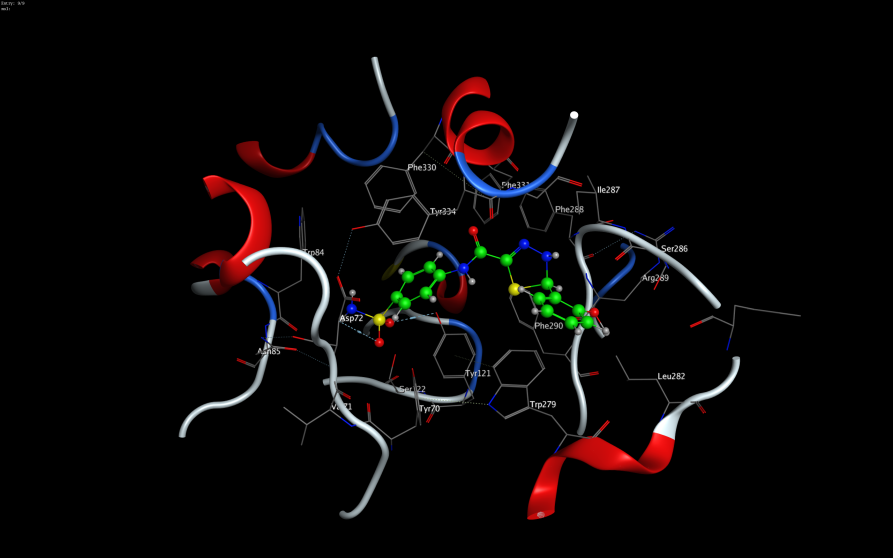 | 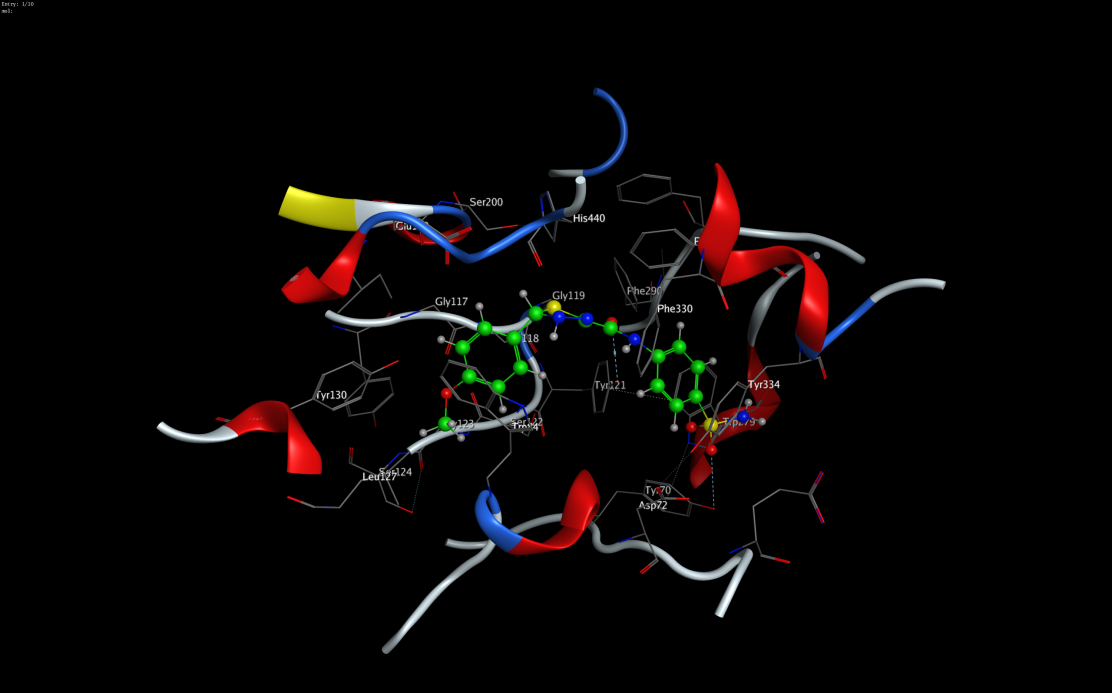 |
| **12** |  |
| 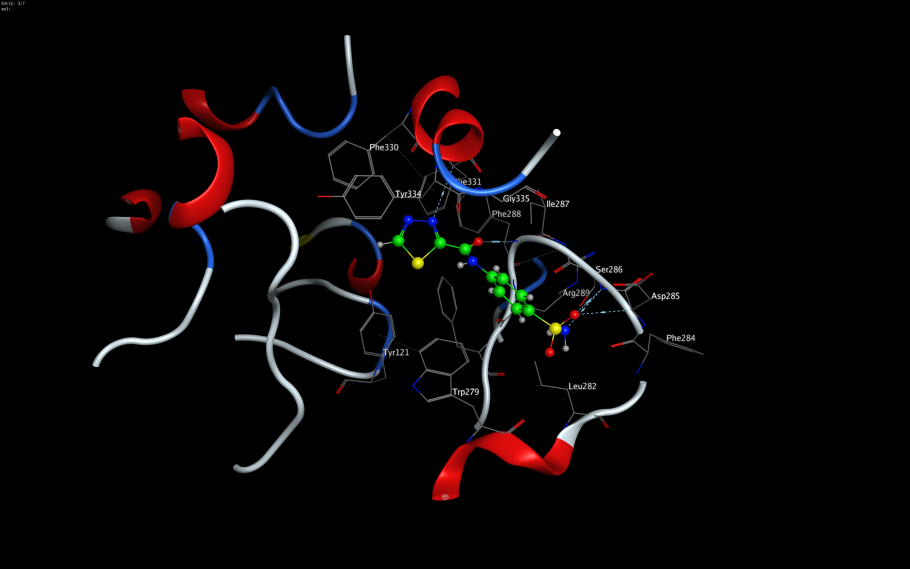 |  |

**Figure S38:** 3D representations of the molecular interactions of the investigated compounds (from compound **2** to compound **12**) against AChE (PDB ID: 2ACE).

| **2** | **3** |
| --- | --- |
| 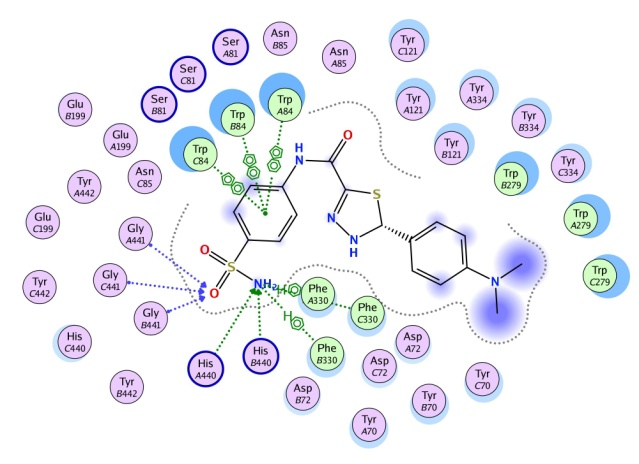 | 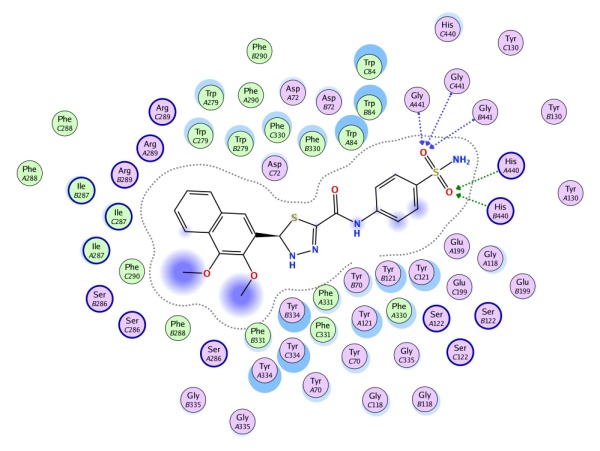 |
| **4** | **5** |
| 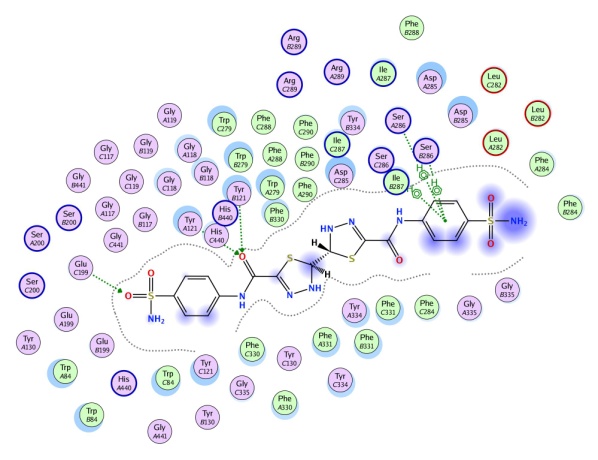 | 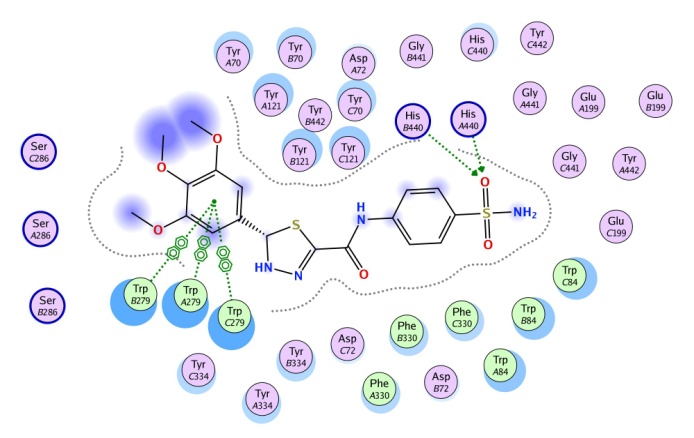 |
| **6** | **7** |
| 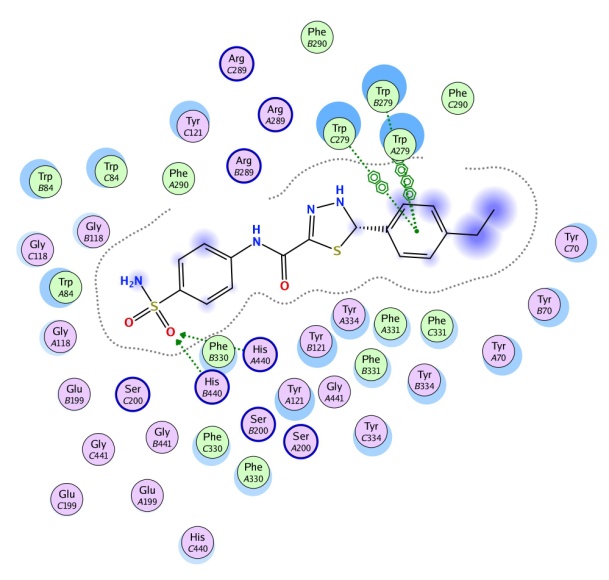 | 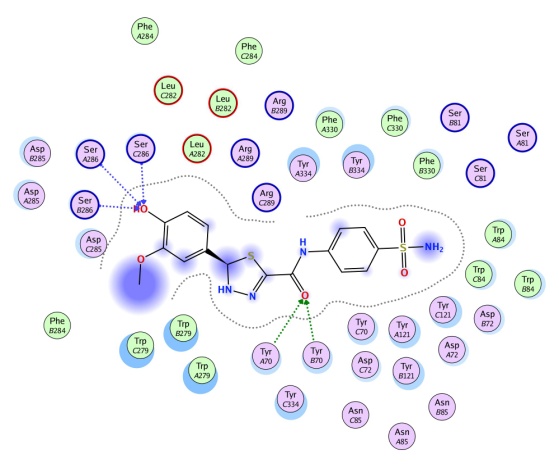 |
| **8** | **9** |
| 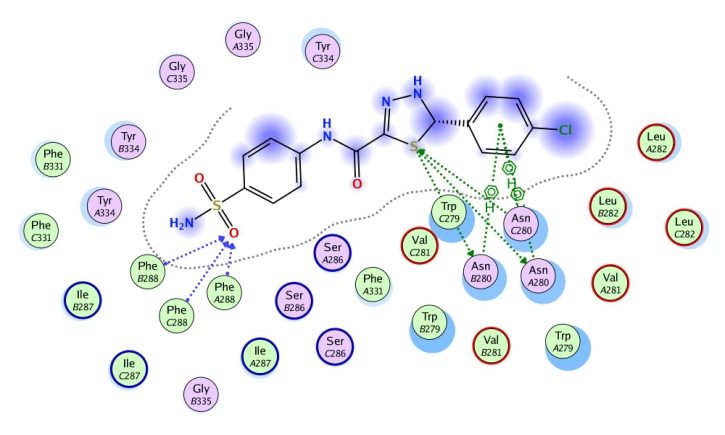 | 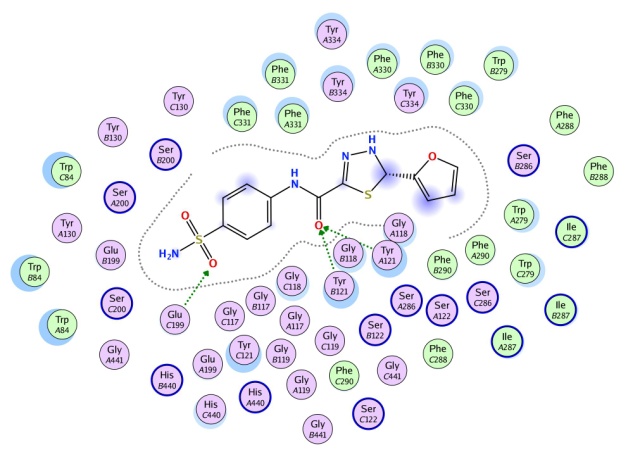 |
| **10** | **11** |
| 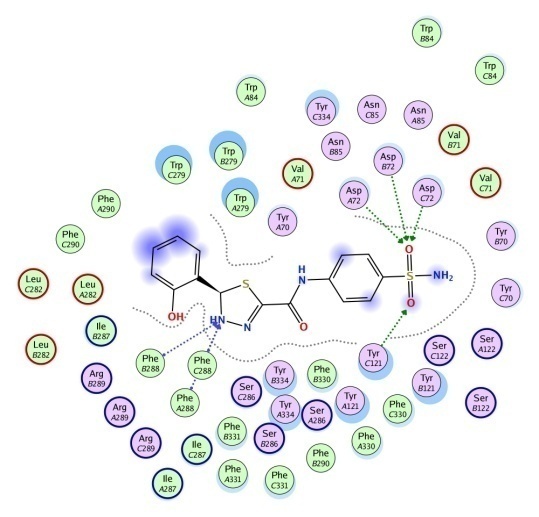 | 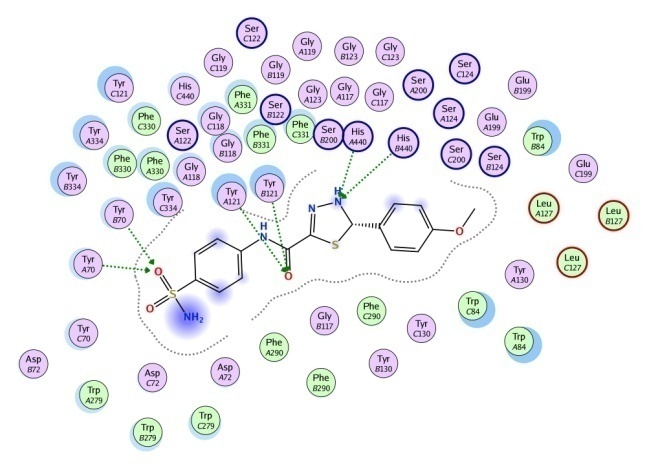 |
| **12** |  |
| 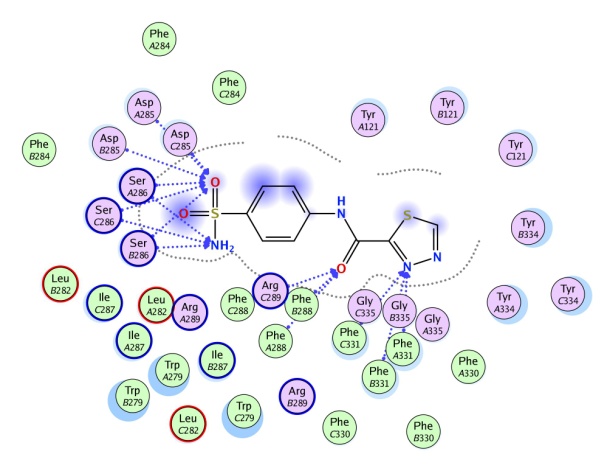 |  |

**Figure S39**: 2D representations of the molecular interactions of the investigated compounds (from compound **2** to compound **12**) against AChE (PDB ID: 2ACE).
